# Serine-linked PARP1 auto-modification controls PARP inhibitor response

**DOI:** 10.1038/s41467-021-24361-9

**Published:** 2021-07-01

**Authors:** Evgeniia Prokhorova, Florian Zobel, Rebecca Smith, Siham Zentout, Ian Gibbs-Seymour, Kira Schützenhofer, Alessandra Peters, Joséphine Groslambert, Valentina Zorzini, Thomas Agnew, John Brognard, Michael L. Nielsen, Dragana Ahel, Sébastien Huet, Marcin J. Suskiewicz, Ivan Ahel

**Affiliations:** 1 Sir William Dunn School of Pathology, University of Oxford, Oxford, UK; 2 Univ Rennes, CNRS, Structure Fédérative de Recherche Biosit, IGDR (Institut de Génétique et Développement de Rennes) – UMR 6290, Rennes, France; 3 Laboratory of Cell and Developmental Signaling, Center for Cancer Research, National Cancer Institute, Frederick, MD USA; 4 Proteomics Program, Novo Nordisk Foundation Center for Protein Research, Faculty of Health and Medical Sciences, University of Copenhagen, Copenhagen, Denmark; 5 Institut Universitaire de France, Paris, France; 6 Present Address: Department of Biochemistry, University of Oxford, Oxford, UK

**Keywords:** DNA damage response, PolyADP-ribosylation

## Abstract

Poly(ADP-ribose) polymerase 1 (PARP1) and PARP2 are recruited and activated by DNA damage, resulting in ADP-ribosylation at numerous sites, both within PARP1 itself and in other proteins. Several PARP1 and PARP2 inhibitors are currently employed in the clinic or undergoing trials for treatment of various cancers. These drugs act primarily by trapping PARP1 on damaged chromatin, which can lead to cell death, especially in cells with DNA repair defects. Although PARP1 trapping is thought to be caused primarily by the catalytic inhibition of PARP-dependent modification, implying that ADP-ribosylation (ADPr) can counteract trapping, it is not known which exact sites are important for this process. Following recent findings that PARP1- or PARP2-mediated modification is predominantly serine-linked, we demonstrate here that serine ADPr plays a vital role in cellular responses to PARP1/PARP2 inhibitors. Specifically, we identify three serine residues within PARP1 (499, 507, and 519) as key sites whose efficient HPF1-dependent modification counters PARP1 trapping and contributes to inhibitor tolerance. Our data implicate genes that encode serine-specific ADPr regulators, HPF1 and ARH3, as potential PARP1/PARP2 inhibitor therapy biomarkers.

## Abstract (web summary)

PARP inhibitors function by trapping PARP1 protein on DNA breaks, which has cytotoxic consequences to cancer cells. Here the authors identify three serine residues within PARP1 as key sites whose efficient HPF1-dependent modification counters PARP1 trapping and contributes to inhibitor tolerance.

## Introduction

Poly(ADP-ribose) polymerase 1 (PARP1) binding to DNA damage stimulates ADP-ribosylation (ADPr) at numerous sites, both within PARP1 itself and in other proteins^1,2^. PARP1 is assisted by two less abundant paralogs that also catalyse ADPr in response to DNA damage, PARP2 and PARP3^3^. The ADPr modification events, including poly(ADP-ribose) (PAR) chains of varying length, facilitate DNA repair by promoting chromatin remodelling and recruitment of DNA repair factors^4–6^. In addition to impairing DNA repair, PARP1/PARP2 inhibitors—which are in clinical use against a growing list of cancers—trap the abundant PARP1 protein on DNA breaks, which has genotoxic cytotoxic consequences, especially in BRCA1- or BRCA2-deficient cancers^7,8^. PARP1 trapping refers to prolonged residence of bulk PARP1 on damaged chromatin, which might result from physical stalling of PARP1 at DNA breaks but could also consist in continuous recruitment and exchange of PARP1 molecules at these sites^9,10^. Persistence of inhibited PARP1 is the reason why PARP1 inhibition is more detrimental than loss of PARP1^8,11,12^. Although some inhibitors have additional allosteric properties^8,13^, the available data suggest that PARP1 trapping is caused primarily by the catalytic inhibition of PARP-mediated ADPr, which is needed to terminate PARP1’s association with, or persistent recruitment to, chromatin^9,14,15^. However, the ADPr sites that contribute to this process remain unknown. While in vitro studies point to the role of PARP1 auto-modification in preventing the interaction with DNA breaks^8,15–18^—presumably through steric and electrostatic interference—it is unclear if auto-modification counteracts trapping in vivo, and, if yes, which exact sites are involved.

With these questions in mind, we turned our attention to serine residues, which we and others have recently revealed to be the main physiological acceptors of ADPr across the human proteome under both basal and DNA-damage conditions, especially if only PARP1- or PARP2-dependent or nuclear ADPr is concerned. The prominence of serine ADPr is reflected both in the number of modification sites detected by mass spectrometry^19–24^ and the amount of ADPr revealed through immunoblotting^24–26^. We previously identified histone PARylation factor 1 (HPF1) as a PARP1 and PARP2 regulator essential for efficient serine modification^20,25,27–29^. Structural analysis revealed that HPF1 completes the PARP active site by providing an additional catalytic residue (Glu284 of HPF1) that dictates robust ADPr initiation reaction at serines^29^. Although a sensitive mass spectrometry approach still detects a number of serine sites in cells lacking HPF1^24^, the loss of HPF1 results in a specific ~200-fold reduction of DNA damage-induced serine ADPr, rendering non-serine ADPr relatively more prominent. Indeed, the major modification events in the absence of HPF1 visualised with anti-ADPr immunoblots appear to be localised to glutamate and aspartate residues, as judged by their hydroxylamine sensitivity^25^. In addition to positive dependence on HPF1, serine ADPr is negatively regulated by the serine-specific ADP-ribosylhydrolase 3 (ARH3)^30,31^, the deletion of which results in an ~100-fold increase in basal modification level of serine sites^24^. Possible other candidates for regulation by the HPF1/ARH3 axis include chemically similar but far rarer ADPr events on tyrosine and threonine residues, although only the connection between the first of them and HPF1 has been established so far^32,33^. Other ADPr types, including glutamate and aspartate and also cysteine, histidine, or arginine attachments, clearly do not correlate with HPF1 and ARH3 presence/loss in the manner in which serine ADPr does^24^. This indicates that they are not regulated by these factors and, indeed, except for acidic residues, most likely are not dependent on PARP1 or PARP2.

Here, we demonstrate that globally altering serine ADPr levels through manipulation of HPF1 or ARH3 levels affects PARP1 residence on chromatin, PARP1/PARP2 inhibitor sensitivity, and inhibitor-mediated synthetic lethality with BRCA1/BRCA2 deficiencies. We also identify serine residues 499, 507 and 519 as the predominant in vivo PARP1 auto-modification sites and demonstrate that preventing their modification through mutation is sufficient to prolong PARP1 retention on DNA damage and sensitise cells to PARP1/PARP2 inhibitors. Altogether, our data connect for the first time PARP trapping and PARP1/PARP2 inhibitor response with specific ADPr sites and implicate HPF1 and ARH3 protein levels as potential biomarkers that predict vulnerability or resistance to therapies based on PARP1/PARP2 inhibition.

## Results

### HPF1 loss enhances PARP1/PARP2 inhibitor sensitivity and PARP-BRCA synthetic lethality

**Fig. 1:**
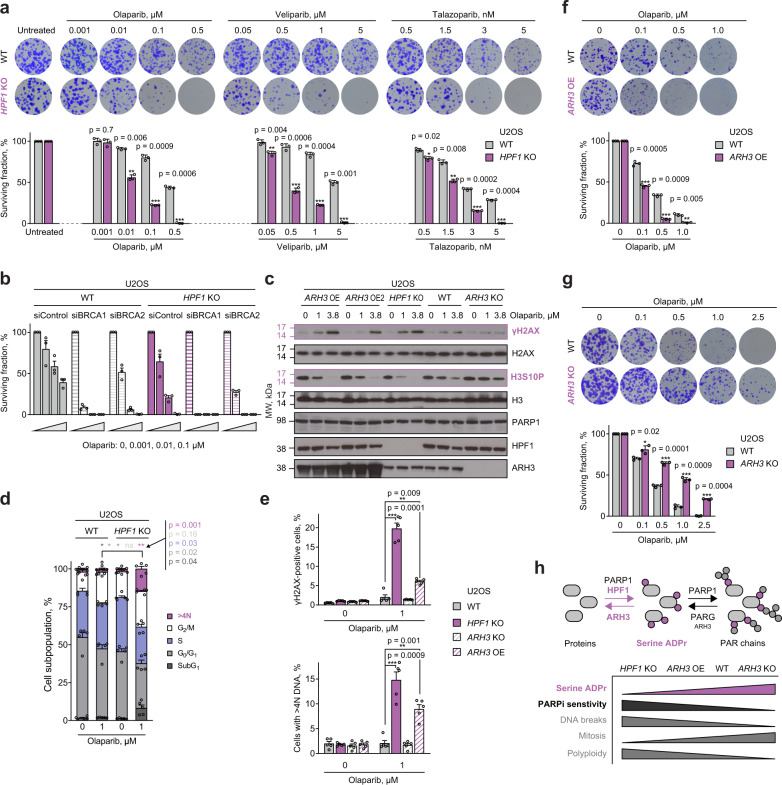
PARP1/PARP2 inhibitor sensitivity is determined by serine ADPr levels, which are controlled by HPF1 and ARH3 activity. **a** Reduced survival of *HPF1* KO cells after treatment with the indicated PARP1/PARP2 inhibitors. Representative images (top) and quantification of colony formation assay (bottom). **b** HPF1 loss results in further sensitisation of BRCA1- or BRCA2-deficient cells to Olaparib. See Supplementary Fig. 2a for BRCA1, BRCA2, HPF1 and tubulin control immunoblots. **c** Effects of 6-day Olaparib treatment on *γ*H2AX formation and H3S10P reduction depend on cellular HPF1 and ARH3 protein levels. The experiment was repeated independently 3 times with similar results. See Supplementary Fig. 2b for Pan ADPr immunoblot. A repeat of the experiment with an additional concentration of Olaparib is provided in Supplementary Fig. 2c. **d** Flow cytometry analysis of cell cycle profiles following 4-day exposure to Olaparib. Asterisks in different colours indicate significant difference in corresponding cell populations between WT and *HPF1* KO cells. **e** HPF1 and ARH3 status determines the effects of 4-day Olaparib treatment on γH2AX levels (top) and percentage of cells with >4 N DNA (bottom) as determined by flow cytometry. **f** *ARH3* overexpression (OE) renders cells more sensitive to PARP1/PARP2 inhibition. **g** Loss of ARH3 confers resistance to Olaparib. **h** Schematic representation of ADPr synthesis and removal (top), and summary of the impact of HPF1 and ARH3 status on cell serine ADPr levels and PARP1/PARP2 inhibitor (PARPi) sensitivity (bottom). **a**, **f**, **g** Data are shown as mean ± SD of three independent experiments. **b**, **d**, **e** Data are shown as mean ± SEM of three (**b**) or five (**d**, **e**) independent experiments. *p < 0.05; **p < 0.01; ***p < 0.001 (two-tailed Student’s *t*-test). Flow cytometry gating strategy for the analyses shown in (**d**) and (**e**) is shown in Supplementary Fig. 2d.

To investigate whether, and through what mechanism, HPF1 and serine ADPr impact the PARP inhibitor response, we first tested sensitivity of cells lacking HPF1 to various PARP inhibitors that differ in their specificity and pharmacological properties. To this end, we used several clinically relevant PARP1- and PARP2-specific inhibitors^34^ (Olaparib, Talazoparib, Veliparib, Niraparib and Rucaparib) and, as a control, compared them with the PARP3 inhibitor ME0328^35^, the PARP5a/PARP5b (TNKS1/TNKS2) inhibitor XAV-939^36^, and the PARG inhibitor PDD00017273^37^. By performing a long-term colony formation assay, we observed marked sensitisation to all PARP1/PARP2 inhibitors in U2OS cells upon the loss of HPF1 (Fig. 1a and Supplementary Fig. 1a). This is consistent with the established role of HPF1 in selectively regulating these two PARPs^27,29^ and our previous observation of *HPF1* knockout (KO) cell sensitivity to Olaparib^27^. The specificity of this effect is confirmed by the fact that *HPF1* deletion did not significantly impact sensitivity to PARP3, PARP5a/b, or PARG inhibitors (Supplementary Fig. 1a–c). Importantly, as shown before^27^, simultaneous deletion of *HPF1* and *PARP1* largely abolished *HPF1* KO sensitivity to Olaparib (Supplementary Fig. 1d), supporting the dependence of the inhibitor-induced survival defect in *HPF1* KO cells on PARP1 inhibition and/or trapping.

Loss-of-function mutations in either *BRCA1* or *BRCA2* lead to defects in double-strand break (DSB) repair by homologous recombination (HR)^38,39^ and also render cells hypersensitive to PARP1/PARP2 inhibition^40,41^. PARP1 plays a key role in single-strand break (SSB) repair^42^ and has been suggested to be retained primarily at SSBs upon PARP1/PARP2 inhibitor treatment^7^. After being encountered by the transcription machinery or a replication fork, DNA-bound PARP1 is thought to trigger DSB formation^7^. These DSBs can then be resolved by functional HR, a major pathway for DSB repair in S-phase, but can only be repaired by alternative, less efficient and error-prone pathways in HR-defective BRCA1*-* or BRCA2*-*deficient cells. Given that above, upon HPF1 loss, we observed a striking cell sensitivity to PARP1/PARP2 inhibition similar to that in HR-defective cells, we next tested whether deleting *HPF1* would enhance the effect of PARP1/PARP2 inhibition on cells with BRCA1 or BRCA2 deficiency. To that end, U2OS WT or *HPF1* KO cells were transfected with control, *BRCA1* or *BRCA2* siRNAs and assessed for survival in response to PARP1/PARP2 inhibition. As expected, knockdown of *BRCA1* or *BRCA2* sensitised WT cells to Olaparib. However, when *BRCA* downregulation was combined with *HPF1* deletion, inhibitor sensitivity was even larger, with complete killing achieved with as little as 1 nM Olaparib for *BRCA1* knockdown (Fig. 1b and Supplementary Fig. 2a). The loss of HPF1 thus enhances the synthetic lethality between PARP1/PARP2 inhibition and BRCA1/BRCA2 deficiency, suggesting that the effect of *HPF1* deletion lies upstream of the deficiencies in HR repair and might involve protection from PARP1 trapping.

Next, we went on to further characterise the phenotypes of HPF1-deficient cells treated with a PARP1/PARP2 inhibitor. Since these experiments were performed in a shorter time frame than that for the survival assays above (where toxic effects of inhibition can accumulate over time), we applied higher Olaparib concentrations. We first observed that the loss of HPF1 results in increased DSB formation (as marked by increased levels of *γ*H2AX) upon Olaparib treatment (Fig. 1c and Supplementary Fig. 2b, c). We also examined the cell cycle distribution and found that the loss of HPF1 in Olaparib-treated cells led to accumulation of endoreplicating polyploid cells with >4 N DNA content, and decreased cell proliferation as shown by reduced EdU incorporation and increased cell death (SubG_1_ subpopulation) (Fig. 1d, e). Under the same conditions, we also detected a much higher fraction of γH2AX-positive cells (Fig. 1e). These effects indicate that HPF1 loss potentiates the PARP1/PARP2 inhibitor-induced DNA damage load and causes problems with fundamental cellular processes of DNA replication, chromatin segregation and cell division. Similar changes have been associated with the cytotoxic effects of PARP1/PARP2 inhibition in different cells^43–45^, which suggests that the absence of HPF1 aggravates these effects rather than triggering separate events.

### Changing serine ADPr levels through ARH3 manipulation modulates PARP1/PARP2 inhibitor sensitivity

HPF1 deficiency dramatically reduces serine-linked ADPr but could also have additional, independent consequences. Therefore, to confirm that PARP1/PARP2 inhibitor sensitivity observed upon *HPF1* deletion is related to the impairment of serine ADPr, we suppressed this modification through alternative means, namely by overexpressing ARH3, the hydrolase that we previously identified as specific for the serine-ADP-ribose linkage^31^. Strikingly, stable *ARH3* overexpression (OE) in U2OS cells, despite having no effect on HPF1 or PARP1 protein levels (Fig. 1c and Supplementary Fig. 2c), resulted in very similar effects to those observed upon *HPF1* KO. These include exacerbated sensitivity to PARP1/PARP2 inhibitors (demonstrated for Olaparib) (Fig. 1f), as well as a marked increase in γH2AX formation (Fig. 1c and Supplementary Fig. 2c) and in the percentage of cells with >4 N DNA content and γH2AX-positive cells (Fig. 1e) upon Olaparib treatment. In addition, we observed a significant decrease in H3 serine 10 phosphorylation levels, a marker of cells that undergo mitosis, in both *HPF1* KO and *ARH3*-overexpressing cells following treatment with Olaparib (Fig. 1c). This suggests that both of these cell lines were unable to enter mitosis and instead continued with DNA replication as manifested by an increased number of polyploid cells.

We hypothesised that if the absence of, or strong reduction in, serine-linked ADPr is a major cause of PARP1/PARP2 inhibitor sensitivity, then, conversely, globally increasing the levels of this post-translational modification might cause PARP1/PARP2 inhibitor resistance. Consistent with this hypothesis, the loss of ARH3 in U2OS cells, which elevates serine-linked ADPr levels^24,25,31^, resulted in a marked resistance to Olaparib when compared to WT cells (Fig. 1g). Moreover, upon Olaparib treatment, these cells showed almost no formation of *γ*H2AX (Fig. 1c), induction of polyploidy (Fig. 1e), or decrease in the levels of mitotic cells (Fig. 1c). Overall, these results strongly support a model whereby PARP1/PARP2 inhibitor susceptibility is controlled by the levels of serine-linked ADPr and wherein HPF1 and ARH3 play indirect roles by regulating this modification (Fig. 1h).

### HPF1-dependent changes in PARP1 auto-modification levels correlate with PARP1/PARP2 inhibitor sensitivity

**Fig. 2:**
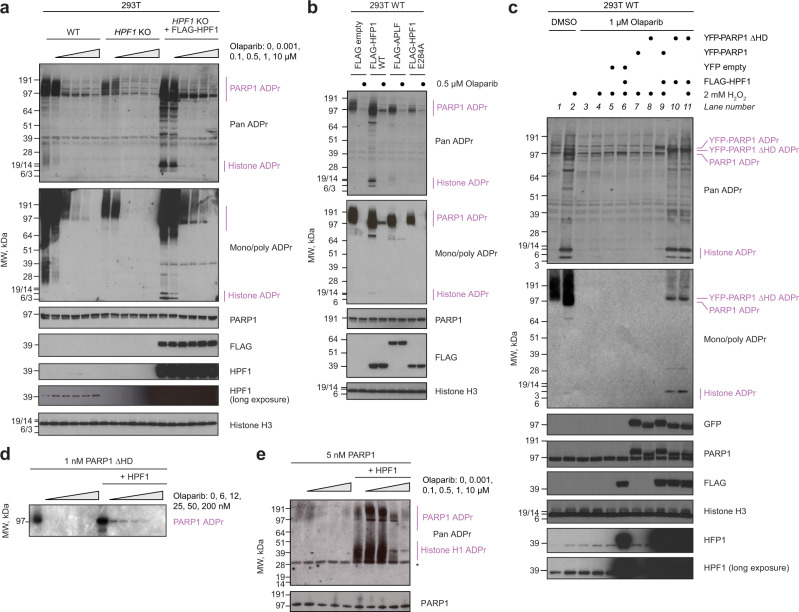
Serine ADPr persists at high doses of PARP1/PARP2 inhibitor. **a** Cellular ADPr levels detected throughout increasing molarities of Olaparib depend on HPF1 status. **b** Overexpression of FLAG-HPF1 WT but not catalytic mutant E284A leads to ADPr that persists despite Olaparib treatment. **c** High ADPr levels achieved by YFP-PARP1 ΔHD overexpression despite Olaparib treatment depend on simultaneous FLAG-HPF1 overexpression. ADPr levels are also increased above basal levels for this Olaparib concentration by DNA damage (H_2_O_2_), FLAG-HPF1 overexpression, or simultaneous YFP-PARP1 and FLAG-HPF1 overexpression. A similar experiment in *PARP1* KO cells is shown in Supplementary Fig. 3b. **d** In vitro radioactive ADPr assay shows ADPr can be detected at higher Olaparib molarities when HPF1 is present in the reaction. **e** In vitro ADPr assay with higher NAD^+^ concentration than in (**d**) and histone H1 as a substrate. Both PARP1 and histone H1 ADPr can be detected at relatively high Olaparib molarities when HPF1 is present. Asterisk indicates non-specific recognition of unmodified histone H1 by the Pan ADPr reagent. In the Pan ADPr blots in (**a**) and (**c**), the weak bands recurrent across all conditions represent a background against which specific signal should be interpreted. **a**–**e** The experiments were performed independently at least 2 times with similar results.

The changes in modification that we triggered by manipulating HPF1 and ARH3 would globally apply to all or most of the thousands of serine (and possibly other hydroxyl) ADPr sites that can be detected in cells. However, the observed sensitivity profiles are most likely related to a (small) subset of these sites that are mechanistically involved in counteracting PARP1 trapping. The well-established role of PARP1 auto-modification in regulating PARP1-DNA interaction in vitro^8,15–18^ points to serine sites within PARP1 as possible candidates. Moreover, PARP1 auto-modification accounts for a major fraction of the total protein-linked ADPr^25^ (Supplementary Fig. 6) and it is more conceivable that bulk behaviour of a very abundant protein such as PARP1 is regulated by an abundant modification. Before directly testing the importance of serine-linked auto-modification for limiting PARP1 trapping and improving PARP1/PARP2 inhibitor tolerance, we first asked if changes in PARP1 auto-modification levels in the presence and absence of HPF1 at various molarities of inhibitors correlate with observed sensitivity profiles. Importantly, PARP1 is significantly auto-modified both in the presence and absence of HPF1. In the latter situation the auto-modification appears to be primarily on glutamate and aspartate residues^25^, although it also includes inefficiently modified serine sites^24^. The existence of such HPF1-independent auto-modification allows the proposal of a unifying mechanism for preventing trapping that could explain why HPF1 deficiency is normally well-tolerated and only becomes toxic when cells are challenged with PARP1/PARP2 inhibition. Consistent with this idea, we hypothesised that various forms of PARP1 auto-modification are functionally equivalent and there might be a minimal threshold of auto-modification that is required for cell survival. If that is the case, then HPF1-independent auto-modification should be brought below this level with lower PARP1/PARP2 inhibitor concentrations compared to HPF1-dependent serine ADPr. To test this model, we monitored ADPr levels in 293T cells with increasing molarities of Olaparib (Fig. 2a). In these cells, endogenous ADPr is more easily detected in the absence of exogenous stimuli compared to U2OS cells^25^, which allowed to perform the experiment without stimulating ADPr using exogenous DNA damaging agents. Whereas for WT cells, ADPr was relatively high in the absence of inhibitors and could be detected on PARP1, as well as histones and other substrates, in *HPF1* KO cells ADPr under the same conditions was markedly lower and appeared limited only to PARP1 auto-modification. Moreover, upon addition of as little as 0.1 µM Olaparib, the ADPr signal in *HPF1* KO cells was no longer detectable. On the other hand, in WT cells ADPr was only fully lost with 10 µM Olaparib. Next, we transiently overexpressed FLAG-tagged HPF1 in *HPF1* KO cells, which increased HPF1 levels far above its normal low cellular abundance. This procedure caused a robust PARP1 auto-modification signal that persisted even at high inhibitor concentrations. Similar effects of manipulating HPF1 levels on PARP1 auto-modification were seen when titrating Talazoparib (Supplementary Fig. 3a). In a follow-up experiment, significant auto-modification of PARP1 in the presence of Olaparib was observed in 293T WT cells when overexpressing WT HPF1 but not HPF1 mutated in the critical catalytic residue, Glu284, or a control PAR-binding protein, APLF (Fig. 2b). Overall, these data demonstrate that the auto-modification synthesised by PARP1 in the presence of catalytically competent HPF1 requires higher inhibitor concentrations to be suppressed compared to auto-modification produced by PARP1 alone, consistent with the increased PARP1/PARP2 inhibitor sensitivity of HPF1-deficient cells.

To further explore this potential mechanism, we probed the effect of exogenous DNA damage and of deleting the autoinhibitory helical subdomain (HD) of PARP1 on the ADPr signal detectable under Olaparib treatment (Fig. 2c). Both damage induction and HD deletion (ΔHD) have a dual consequence: they activate PARP1^46^ while also enhancing its affinity for HPF1 (which is relatively low for PARP1’s unactivated form^29^). We monitored ADPr in 293T WT cells in the presence of 1 µM Olaparib, a concentration that in the above Olaparib-titration experiment led to a very low, but still detectable PARP1 auto-modification signal without any overexpressed proteins. We observed that this signal, detected especially with anti-Pan ADPr reagent, could be increased slightly by hydrogen peroxide (H_2_O_2_)-induced DNA damage (lane 4), and further by simultaneous transient overexpression of FLAG-tagged HPF1 (lane 6), consistent with the low endogenous levels of HPF1 being the limiting factor^27^. Conversely, overexpressing YFP-PARP1, either WT or ΔHD, without simultaneous HPF1 co-expression did not result in any additional signal compared to DNA damage alone (lanes 7 and 8), despite the fact that PARP1 ΔHD is constitutively hyperactive in vitro^46^. However, when FLAG-HPF1 was simultaneously co-expressed with WT or ΔHD YFP-PARP1, the resultant HPF1-PARP1 complexes produced significant auto-modification despite high Olaparib concentration (lanes 9–11). Artificially improving PARP1 binding to HPF1 through HD deletion leads to more complete saturation of PARP1 with HPF1^29^, which—combined with a possible higher activity of this mutant—could explain a particularly striking effect with PARP1 ΔHD. Similar results were obtained in 293T PARP1 KO cells (Supplementary Fig. 3b), excluding the possibility that the presence of endogenous PAPR1 could affect the results above. Overall, these data support a model in which PARP1 in combination with HPF1 is able to produce PARP1 auto-modification levels that are still significant at much higher doses of PARP1 inhibitor compared to PARP1 alone.

### HPF1 counteracts PARP1/PARP2 inhibition by stimulating PARP1 auto-modification

The difference in abundance of HPF1-dependent and -independent PARP1 auto-modification in untreated cells and under inhibitor treatment could result from intrinsic properties of PARP1 with and without HPF1, or could be related to how these different ADPr types are processed by cellular hydrolases. For example, a modification that is hydrolysed to a smaller extent would be more abundant and thus require more inhibitor to be suppressed even if it was synthesised with equal efficiency. To approach this question, we performed in vitro ADPr assays looking at the initial stage of the PARP1 auto-modification reaction in the presence or absence of HPF1 and with increasing molarities of Olaparib but without any additional factors, such as hydrolases, that would be present in the cells. We tested separately the PARP1 ΔHD-HPF1 complex (using radioactivity-based detection, Fig. 2d) and the full-length PARP1-HPF1 complex in the presence of histone H1 as an additional substrate (using anti-Pan ADPr reagent-based detection, Fig. 2e). Consistent with in vivo results, both PARP1 auto-modification and trans-modification of a histone substrate were stimulated by HPF1 in vitro. Moreover, ADPr produced in the presence of HPF1 was detectable at higher Olaparib concentrations. These results are consistent with in vivo data presented above and suggest that the observed effect is at least partially independent of hydrolysis and is related to how PARP1 synthesises ADPr when assisted by HPF1 or acting alone.

The fact that HPF1-dependent PARP1 auto-modification, compared to its HPF1-independent form, requires higher levels of inhibitor to be suppressed could be explained in two non-mutually exclusive ways. In the first scenario, it might be due to higher abundance of this modification. Indeed, we showed above that this is the case in vivo and can be attributed at least in part to higher efficiency of synthesis. Because PARP1 is more active when assisted by HPF1, even when inhibited to the same relative extent by a given amount of inhibitor, it produces more modification. Additionally, the observed effect could be amplified by changes in binding properties of PARP1 in the presence of HPF1: decreased affinity for inhibitors and/or increased affinity for the substrate NAD^+^, both of which would result in inhibitors being relatively disfavoured in their competition with NAD^+^. In this second scenario, the same amount of inhibitor would decrease the starting activity of PARP1 to a lower relative extent in the presence of HPF1 compared to its absence. To explore this additional possibility, we developed a fluorescence anisotropy-based binding assay. First, we characterised the binding of the fluorescent Olaparib derivative, PARPi-FL^47^, to PARP1 in the presence and absence of HPF1. As the binding proved too strong for directly measuring the dissociation constant (*K*_D_) in an equilibrium binding experiment, we separately analysed the association (Supplementary Fig. 4a–c) and dissociation (Supplementary Fig. 4d) kinetics and calculated *K*_D_ using rate constants (Supplementary Table 1**)**. This approach was the same as that recently published^48^ and led to similar results, indicating that PARPi-FL binds similarly or slightly tighter to PARP1-HPF1 compared to PARP1 alone. Subsequently, we performed equilibrium competition assays, in which PARPi-FL was displaced by increasing molarities of Olaparib or Talazoparib (Supplementary Fig. 4e and Supplementary Table 2). Our results indicate that these inhibitors, too, bind similarly or slightly tighter to the PARP1-HPF1 complex compared to free PARP1, in line with values obtained by the Luger group using a different method^48^. Combined with the recent report of similar Michaelis constant (*K*_M_) values of PARP1 for NAD^+^ in the presence and absence HPF1^49^, these results speak against altered PARP1 binding properties as an explanation for the apparent inhibitor resistance seen in our experiments in the presence of HPF1. This effect appears to be primarily attributable to the high efficiency with which serine ADPr is synthesised by the PARP1-HPF1 complex, allowing significant levels of modification to break through high inhibitor concentrations.

### HPF1-dependent serine-linked PARP1 auto-modification counteracts PARP1 trapping

**Fig. 3:**
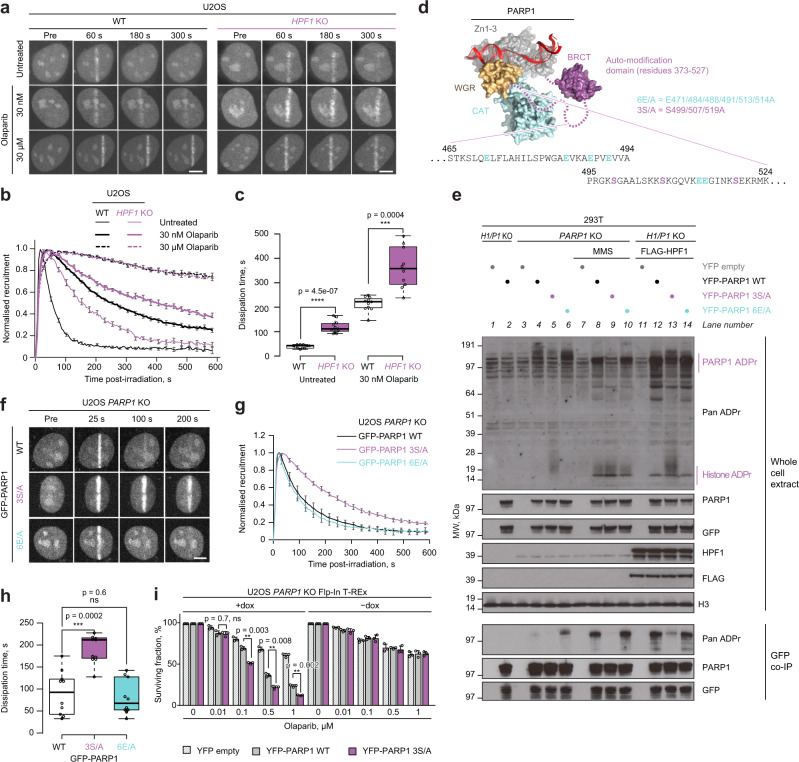
PARP1 function and release from DNA damage depend on serine ADPr. **a** Representative confocal images of U2OS WT or *HPF1* KO cells transiently expressing GFP-tagged PARP1 chromobody to follow endogenous PARP1 recruitment to sites of laser microirradiation over time in the presence of Olaparib. **b** Quantification of GFP-PARP1 chromobody accumulation kinetics at damaged sites in WT and *HPF1* KO cells. A repeat of the experiment with additional concentrations of Olaparib is provided in Supplementary Fig. 5a. **c** Dissipation time of GFP-PARP1 chromobody, corresponding to the time required to dissipate 50% of the maximum PARP1 signal. **d** Model of PARP1 (surface representation coloured according to domain composition) bound to a single-strand DNA break (red ribbon) created by alignment of structures from PDB accessions 4DQY, 2N8A and 2LE0. The auto-modification fragment missing from the structures is shown schematically and its sequence containing ADPr sites mutated in point (**e**) is provided. **e** Mutation in key serine (S499/507/519A, 3S/A) but not glutamate PARP1 auto-modification sites (E471/484/488/491/513/514A, 6E/A) reduces the levels of endogenous and DNA damage (MMS)- or FLAG-HPF1 overexpression-induced PARP1 ADPr but increases the levels of histone ADPr. *P1/H1* KO corresponds to *PARP1/HPF1* KO cells. Representative immunoblots of whole-cell extracts and GFP coimmunoprecipitation (GFP co-IP) samples from three independent experiments are shown. **f** Representative confocal images of GFP-PARP1 WT, 3S/A or 6E/A recruitment to sites of laser microirradiation over time. **g** Quantification of the accumulation kinetics of GFP-PARP1 WT, 3S/A or 6E/A at damaged sites. **h** Dissipation time of GFP-PARP1 WT, 3S/A or 6E/A. **i** Loss of key serine auto-modification sites in PARP1 3S/A mutant leads to increased cell sensitivity to Olaparib. Data are shown as mean ± SEM of three independent experiments. See Supplementary Fig. 7b for GFP, PARP1 and H3 control immunoblots. In (**a**) and (**f**), the scale bar represents 5 μm. In (**b**) and (**g**), individual curves were normalised to maximum recruitment; data are shown as mean ± SEM from the analysis of at least 10 nuclei. In (**c**) and (**h**), the box limits correspond to the 25th and 75th percentiles and the bold line indicates the median value; the whiskers extend 1.5 times the interquartile range. *p < 0.05; **p < 0.01; ***p < 0.001 (two-tailed Student’s *t*-test). Experiments shown in (**a**, **e**, **f**) were repeated independently at least 3 times with similar results.

We next turned to studying the importance of serine ADPr and specifically serine-linked PARP1 auto-modification for preventing PARP1 trapping. To directly test if serine ADPr, first considered globally and not on specific sites, contributes to the detachment of PARP1 from DNA damage sites, we analysed the transient accumulation of PARP1 at damaged chromatin in the presence or absence of HPF1. By tracking endogenous PARP1 using GFP-tagged PARP1 chromobody in live-cell microscopy, we demonstrated markedly slower dissociation of PARP1 from laser-irradiated chromatin in *HPF1* KO compared to WT U2OS cells (Fig. 3a–c and Supplementary Fig. 5a). This observation is consistent with a previous result obtained using the ectopically expressed YFP-tagged PARP1^27^. An HPF1-dependent difference in PARP1 dissociation kinetics was also clearly observed in the presence of 30 and 100 nM Olaparib, which falls in the intermediate concentration range for which in the above experiments HPF1 boosted cell survival and prevented complete suppression of PARP1 auto-modification. Performing this analysis with 10 or 30 µM Olaparib, very high concentrations that are expected to fully inhibit PARP1 independently of the HPF1 status, led to the same effect in both *HPF1* KO and WT cells, with PARP1 remaining associated with damage. Similar results were observed when testing intermediate and high Talazoparib concentrations (Supplementary Fig. 5b). These data show that, in the absence of HPF1, PARP1 is more slowly mobilised both without PARP1/PARP2 inhibitors and with clinically relevant intermediate doses of these drugs. Considering the toxic consequences of PARP1 trapping, these differences between WT and *HPF1* KO cells could explain the dramatic sensitivity of the latter to PARP1 inhibitors.

Subsequently, we went on to test the relevance of various PARP1 auto-modification sites for preventing PARP1 trapping, focusing mainly on those in the auto-modification domain, which encompasses the central BRCT domain and a neighbouring unstructured region (Fig. 3d). Before performing live-cell imagining, we characterised which sites account for the largest fraction of PARP1 auto-modification detected with immunoblotting. While there are multiple sites within this segment that have been detected through mass spectrometry^20,23,50,51^ or can be predicted, the importance of none of them has been analysed through mutational studies. We focused on three serine (499, 507 and 519) and six glutamate residues (471, 484, 488, 491, 513 and 514), exploring their relevance through alanine substitution (Fig. 3e). We first transiently expressed WT, S499/507/519A (3S/A), and E471/484/488/491/513/514A (6E/A) YFP-tagged PARP1 variants in U2OS *PARP1* KO cells and monitored ADPr at basal conditions, following exposure to a DNA-damaging agent methyl methanesulfonate (MMS), or upon FLAG-HPF1 overexpression. In all these conditions, the auto-modification signal was relatively similar in cells complemented with WT and 6E/A PARP1 but not in those expressing the 3S/A mutant, which showed a marked decrease in auto-modification. This indicates that the overwhelming amount of PARP1 auto-modification at both basal and DNA-damage conditions locates to these three serine sites and that it is mostly modification of these three sites that is induced in the presence of HPF1. This result is consistent with our previous observation that a major fraction of protein ADPr, including PARP1 auto-modification, is HPF1-dependent^25^. All three PARP1 variants are active as demonstrated by their ability to modify histones, with cells overexpressing PARP1 3S/A even showing higher histone ADPr levels, possibly because more NAD^+^ is available for this reaction upon loss of the major PARP1 sites. A further complementation experiment with combinatorial double PARP1 serine mutations, S499/507A, S499/519A and S507/519A, demonstrated that the first two sites are the most prominent, with their loss producing a similar auto-modification defect to that seen for the 3S/A mutant (Supplementary Fig. 7a). We next asked if abolishing the key serine auto-modification sites is sufficient to enhance PARP1 trapping in HPF1-proficient cells. We conducted this experiment in laser-irradiated U2OS *PARP1* KO cells by tracking GFP-tagged PARP1 WT, 3S/A, or 6E/A using live-cell microscopy in the absence of inhibitors (Fig. 3f). Consistent with the primary importance of the serine sites, the 3S/A mutation, but not the 6E/A one, resulted in an impaired mobilisation compared to WT (Fig. 3g, h). As a control, we also mutated a previously identified tyrosine ADPr site within PARP1 (Y634A) and tested its residence at DNA damage, observing no impact on dissociation kinetics (Supplementary Fig. 5c). Finally, to compare the effects of global serine ADPr loss through *HPF1* deletion and targeted ablation of the identified serine PARP1 auto-modification sites in the same experiment, we used live-cell microscopy to monitor recruitment of WT or 3S/A GFP-PARP1 to DNA damage in laser-irradiated U2OS *PARP1* KO or double *PARP1*/*HPF1* KO cells without inhibitors (Supplementary Fig. 5d). Mobilisation of WT GFP-PARP1 from damage in HPF1-deficient cells was more impaired than that of 3S/A GFP-PARP1 in HPF1-proficient cells, suggesting that while the modification of PARP1 serines 499, 507 and 519 makes a major contribution to preventing PARP1 trapping, there are further HPF1-dependent sites—in or outside PARP1—that also contribute to this process. It is also worth noting that the HPF1-deficient cells show similar trapping of WT and 3S/A PARP1, confirming that the three identified serine sites are mostly HPF1-dependent.

### Loss of serine-linked PARP1 auto-modification sensitises cells to PARP1/PARP2 inhibitors

In the final stage of this study, we asked whether the identified serine PARP1 auto-modification sites contribute to cellular PARP1/PARP2 inhibitor tolerance. To that end, we complemented U2OS *PARP1* KO Flp-In T-REx cells with stably integrated genes encoding YFP-tagged PARP1 (WT or 3S/A) or YFP-only control under doxycycline-inducible promoters (Fig. 3i and Supplementary Fig. 7b). Consistent with previous observations that sensitivity to Olaparib is PARP1-dependent^8^, the cells complemented with YFP alone or with any of the PARP1 variants but in the absence of doxycycline were highly resistant to the inhibitor. Induction of PARP1 expression resembling endogenous expression levels resulted in increased Olaparib sensitivity. Importantly, this effect was markedly stronger when PARP1 3S/A was expressed instead of the WT enzyme. Additionally, we monitored γH2AX levels as a marker for DSBs in these cells. Similarly to what was seen above for cells with globally decreased ADPr, expression of the auto-modification-deficient PARP1 led to higher levels of DNA damage induced by Olaparib treatment (Supplementary Fig. 7c**)**. These findings further support the protective roles of the identified serine sites upon PARP1/PARP2 inhibitor treatment.

Together, our results demonstrate the importance of serine-linked ADPr for limiting PARP1 trapping and PARP1/PARP2 inhibitor-induced toxicity. A substantial part of that effect can be attributed to three prominent auto-modification sites, serines 499, 507 and 519, which, when mutated, increase PARP1’s residence on chromatin and sensitise cells to its inhibitors. To our knowledge, this is the first demonstration of the impact of specific ADPr sites on these processes.

## Discussion

Following the recent identification of protein serine residues as the main physiological acceptor of ADPr^22,25^ and of HPF1 as a specificity factor that is required to efficiently catalyse this process^20,27^, we now demonstrate the crucial importance of HPF1-dependent serine-linked ADPr—specifically on PARP1 itself—for the PARP1/PARP2 inhibitor response. Our data are consistent with a model whereby cell viability requires a minimal threshold of PARP1 auto-modification, which is more robustly maintained when efficient ADPr attachment to serine residues is enabled by appropriate HPF1 and ARH3 levels. Although PARP1 and PARP2 can still catalyse auto-modification in the absence of HPF1, the chains are then inefficiently initiated (presumably primarily on glutamate and aspartate residues), leading to substantially lower ADPr levels in the absence of inhibitors and to easier suppression in their presence. Of note, in vitro studies with free PARP1 suggest that the initial ADP-ribose attachment in the absence of HPF1 is markedly slower than chain elongation and constitutes the rate-limiting step of the process^52^. HPF1, although presumably acting only at the initiation (i.e. mono(ADP-ribosyl)ation or MAR) level, could therefore also increase the PAR synthesis rate by allowing fast attachments to serine residues that can then, in some cases, be robustly extended by PARP1 or PARP2 alone. Here, we did not investigate the relative importance of mono-, oligo- and poly-ADPr for the PARP1/PARP2 inhibitor response, but it is likely that HPF1 will affect, directly or indirectly, all these processes.

Due to its abundance and high affinity for various types of DNA breaks^1^ and obstructions that arise during DNA replication, such as stalled or collapsed replication forks^53^, PARP1 is at a constant risk of getting trapped on chromatin. This points to a key role for a negative feed-back loop whereby PARP1 activation by DNA breaks leads to ADPr, which in turn triggers timely mobilisation of PARP1 from these breaks. Such a cycle has been proposed before based on in vitro studies, which pointed specifically to PARP1 auto-modification as a mechanism that prevented prolonged association of PARP1 with DNA breaks, presumably through electrostatic interference^16^. According to this model, PARP1/PARP2 inhibitors would cause PARP1 trapping and the accompanying toxic consequences primarily by interfering with PARP1’s capacity to self-detach through ADPr. However, it has been unclear to what extent the trapping and detrapping observed in cells—processes the nature of which is still poorly understood^9^—are related to the changes in the direct PARP1-DNA interaction measured in vitro. Here, we directly demonstrate for the first time the importance of auto-modification at specific PARP1 sites for counteracting PARP1 trapping and inhibitor-induced toxicity in vivo. While this seems to support the relevance of the negative feed-back loop model, it is likely that PARP1 auto-modification in cells exerts its protective role not (only) through direct electrostatic or steric effects on DNA binding, but (also) by recruitment of factors that promote PARP1 mobilisation through chromatin remodelling^10^, repair of DNA lesions^9^, and possibly other mechanisms.

Although our data support specifically the importance of ADPr on serines 499, 507 and 519 of PARP1, we suspect that the different serine-linked and other (presumably mostly glutamate/aspartate-linked) auto-modification events are partially redundant in their roles. This redundancy—with most known serine and glutamate/aspartate sites localising to the same auto-modification segment of PARP1—has likely emerged in evolution to ensure that PARP1 dissociation is a robust process that is not easily compromised by point mutations or variations in HPF1 levels. However, when the system is challenged with PARP1/PARP2 inhibitors, the primary importance of the identified three major serine sites comes into light. The fact that glutamate/aspartate ADPr evolved as a ‘backup’ rather than the main form of PARP1 auto-modification might be related to its less efficient modification and lower chemical stability.

**Fig. 4:**
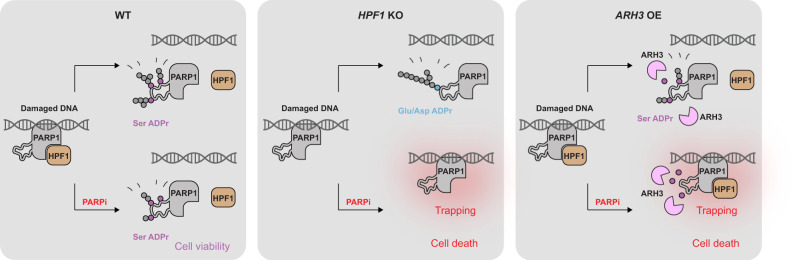
Proposed model of the effect of HPF1 deficiency and ARH3 overexpression on PARP1/PARP2 inhibitor response. In WT cells, HPF1 allows efficient PARP1 auto-modification at serine residues, especially 499, 507 and 519. This modification is more efficiently produced and therefore partially persists at higher inhibitor concentrations compared to HPF1-independent auto-modification (presumably primarily at glutamate or aspartate residues). In the presence of PARP1/PARP2 inhibitors (PARPi), HPF1-dependent serine auto-modification partially escapes inhibition and promotes cell viability by counteracting PARP1 trapping. This mechanism is compromised in *HPF1* KO cells (due to the loss of efficient serine ADPr synthesis) and *ARH3* OE (due to higher levels of serine ADPr hydrolysis), leading to PARP1 trapping and cell death upon inhibitor treatment. Endogenous levels of ARH3 in WT and *HPF1* KO cells are not indicated.

Following recent additional approvals, PARP1/PARP2 inhibitors are currently used against breast, ovarian, pancreatic, and prostate cancers. The important message from this development is that therapeutic benefit has been increased more by identifying new susceptible cancer targets for existing PARP1/PARP2 inhibitors than by developing novel, improved inhibitors. Indeed, the initial designation of these compounds to *BRCA1*/*BRCA2*-mutated cancers already stemmed from the discovery of the synthetic lethal relationship between PARP1/PARP2 inhibition and faulty HR machinery^40,41^. Similarly, our demonstration of synthetic lethality between PARP1/PARP2 inhibition and both HPF1 deficiency and *ARH3* OE points to further genetic backgrounds that would sensitise cells to these therapeutics and implicates HPF1 and ARH3 as potential biomarkers for sensitivity/resistance (Fig. 4). In the case of HPF1, one must bear in mind that rather than simply stimulating PARP1, it modulates its specificity, allowing faster initiation at serine residues, while at the same time, as demonstrated previously^27,29^, limiting excessive chain elongation. This suggests that a correct dosage of HPF1 is important for proper balancing of various PARP1- and PARP2-catalysed reactions, and therefore subtler changes beyond simple loss-of-function might also be of therapeutic relevance in both cancer and other diseases. Of note, in recent screens, HPF1 was detected as one of the top sensitisers, upon deletion, to PARP1/PARP2 inhibitors^54,55^, confirming our previous^27^ and new findings. The dramatic susceptibility observed by us for cells with combined HPF1 and BRCA1/BRCA2 deficiencies indicates that PARP1/PARP2 inhibitor therapy might be particularly successful when changes in HPF1 or ARH3 co-exist with other dysfunctions in DNA repair pathways. Importantly, defects in DNA repair are widespread in cancer, with as many as 50% of ovarian tumours having impaired HR^56^.

Recently, cellular ADPr levels have been proposed as a biomarker that *positively* correlates with PARP1/PARP2 inhibitor sensitivity across certain types of cancer cells^57–59^. It is likely, however, that this correlation—which appears to contradict the protective role of ADPr—indirectly reflects the dependence of inhibitor sensitivity on high PARP1 protein levels and accumulated DNA damage, both of which would manifest as high ADPr levels but enhance PARP1 trapping. We show that if the levels of PARP1 and DNA damage are kept constant by using the same genetic background, ADPr levels—modulated through manipulating secondary ADPr regulators rather than PARP1 protein levels or genome quality—correlate *negatively* with inhibitor sensitivity, consistent with the model proposed above. The protective role of ADPr levels clearly emphasised by our study explains the recently reported increased PARP1/PARP2 inhibitor resistance upon downregulation of the hydrolase PARG^54,60^. Although PARG, unlike ARH3, does not remove the initial attachment on serine residues, it counteracts ADPr synthesis by reversing PAR chain elongation^31,61^.

Our study highlights the embeddedness of PARP1 within a broader network that includes HPF1, ARH3, and PARG, each of which influences PARP1 functions including the negative feed-back loop that prevents its toxic trapping on chromatin. Understanding the complex dynamics within this system and the underlying mechanisms is crucial for making the most of the existing, and developing novel, PARP1/PARP2 inhibitor-based therapies.

## Methods

### Cell lines

Human U2OS osteosarcoma (ATCC HTB-96) and embryonic kidney 293T (ATCC CRL-3216) cells were acquired from ATCC. U2OS *HPF1* KO, U2OS *PARP1* KO, U2OS *HPF1/PARP1* KO, 293T *HPF1* KO, 293T *HPF1/PARP1* KO^27^ and U2OS *ARH3* KO^31^ cells were generated previously. The cells were grown in DMEM (Sigma-Aldrich) supplemented with 10% FBS (Gibco) and penicillin-streptomycin (100 U/mL, Gibco) at 37 °C with 5% CO_2_. U2OS Flp-In T-REx cells were generated by Daniel Durocher’s laboratory^62^; 293T *PAPR1* KO and U2OS Flp-In T-REx *PARP1* KO cells were generated using CRISPR Cas9 genome editing as previously described^16^, using guide RNA sequences complementary to exon 2, PARP-1-sgRNA#1 **(**CCACCTCAACGTCAGGGTG) and PARP-1-sgRNA#2 (TGGGTTCTCTGAGCTTCGT), and the pSpCas9n(BB)-2A-Puro (PX462) V2.0 vector from Feng Zhang (Addgene plasmid #62987). The cells were grown in DMEM supplemented with 10% FBS, 50 µg/mL Zeocin (R25001, Thermo Fisher) and 4 µg/mL Blasticidin (ant-bl-1, Invivogen) at 37 °C with 5% CO_2_.

To generate stable cell lines with *ARH3* OE, U2OS cells were plated in 6 cm dishes and transiently transfected with pDEST12.2 *ARH3* WT using TransIT-LT1 Transfection Reagent (Mirus Bio) according to the manufacturer’s protocol. After 24 h, the cells were transferred into 15 cm dishes and allowed to grow for 48 h. Then, the media was replaced with complete DMEM supplemented with 1 mg/mL G-148 solution (Sigma-Aldrich) for 10 days to select for resistant cells integrated with the pDEST12.2 *ARH3* constructs. Individual colonies were picked using cloning discs (Sigma-Aldrich), propagated and screened to check for successful integration via PCR. Immunoblotting was performed on positive colonies to check ARH3 protein levels.

To generate *PARP1*-expressing inducible cell lines, U2OS Flp-In T-REx *PARP1* KO cells were plated in 6-well plates and cotransfected with YFP empty vector or plasmids encoding YFP-PARP1 WT or 3S/A and the Flp recombinase plasmid pOG44 (in 1:9 ratio) using TransIT-LT1 Transfection Reagent (Mirus Bio) according to the manufacturer’s protocol. At 24 h following transfection, cells were transferred into 15 cm dishes in DMEM with 10% FBS; 24 h later the media was supplemented with 4 µg/mL Blasticidin (Invivogen) and 200 µg/mL Hygromycin B Gold (Invivogen). The media in the dishes was subsequently changed every 2–3 days for two weeks to select for resistant colonies, which were picked using cloning discs (Sigma-Aldrich), propagated and screened following 24 h incubation with 1 µg/mL doxycycline (Sigma-Aldrich) by fluorescence microscopy and immunoblotting. To resemble endogenous PARP1 protein expression levels in the selected clones, YFP empty vector and YFP-PARP1 expression was induced with 0.1 µg/mL doxycycline; YFP-PARP1 3S/A expression was induced with 0.5 µg/mL doxycycline. The media was replenished every 3 days to maintain expression levels in long-term assays.

### Chemical compounds

PARP inhibitors Olaparib, Talazoparib and Veliparib were purchased from Cayman Chemical or Enzo Life Sciences; Niraparib, Rucaparib and ME0328 from Stratech Scientific; XAV-939 and PDD00017273 from Sigma-Aldrich, and were dissolved in dimethyl sulfoxide (DMSO) (Sigma-Aldrich). Methyl methanesulfonate (MMS) and hydrogen peroxide (H_2_O_2_) were obtained from Sigma-Aldrich. Concentrations and durations of treatment are indicated in the sections below and in the respective figures.

### Colony formation assay

Cells were plated at low densities (700 cells/well for U2OS cells and 900 cells/well for U2OS Flp-In T-REx cells) in 6-well plates and grown in the indicated conditions for 11 days. Cells were fixed and stained with 0.5% crystal violet in 25% methanol for 30 min, washed with water and air-dried. Quantification was performed using ImageJ software. The surviving fraction at each dose was calculated after normalisation to the plating efficiency of untreated samples.

### siRNA transfection

siRNA transfection was performed using Lipofectamine RNAiMAX (Invitrogen) according to the manufacturer’s instructions. Silencer Select Negative Control No. 2 siRNA and siBRCA1 (s458, CAGCUACCCUUCCAUCAUA) were from Ambion (Invitrogen). siBRCA2 (D-003462-04, GAAGAAUGCAGGUUUAAUA) was purchased from Dharmacon.

### Cell cycle analysis

Cells were seeded in 6-well plates, treated and incubated with 10 µM EdU for 1 h at the end of treatment. Cells were harvested by trypsinization and labelled using the Click-iT Plus EdU Alexa Fluor 647 Flow Cytometry Assay Kit (Invitrogen) according to the manufacturer’s instructions. For the analysis of DSB levels, cells were then stained protected from light with γH2AX primary antibody (Cell Signaling, 9718S, 1:200) in 1% BSA in PBS for 1 h at room temperature, washed once and incubated for 30 min with Alexa Fluor 488-conjugated goat anti-rabbit secondary antibody (Molecular Probes/Thermo Fisher, A11034, 1:500) in 1% BSA in PBS. For DAPI staining, cell pellets were resuspended in 1 μg/mL DAPI solution in PBS and incubated for 10 min. Cells were washed in PBS and analysed immediately after staining on Cytoflex LX (Beckman Coulter), using CytExpert version 2.3 (Beckman Coulter) for data collection. Post-acquisition analysis was performed in FlowJo software version 10 (BD Biosciences).

### Plasmids and site-directed mutagenesis

Vectors for bacterial expression of full-length human HPF1 and PARP1 with N-terminal FLAG or YFP tags were previously described^27^. Mammalian expression vectors encoding FLAG-HPF1 E284A and YFP-PARP1 ΔHD^29^, pmEGFP-PARP1^10^, and FLAG-APLF^63^ were generated previously. PARP1 point mutations were introduced through site-directed mutagenesis PCR using the QuickChange Lightning kit (Agilent). The primers used to introduce these mutations are listed in Supplementary Table 3.

### Immunoblotting

The cells were lysed with Triton-X100 lysis buffer (50 mM Tris-HCl pH 8.0, 100 mM NaCl, 1% Triton X-100) supplemented with 5 mM MgCl_2_, protease and phosphatase inhibitors (Roche), Olaparib (Cayman Chemical, 1 μM for U2OS and 2 μM for 293T cells) and 1 μM PARG inhibitor PDD00017273 (Sigma-Aldrich, 1 μM for U2OS and 2 μM for 293T cells) at 4 °C. The lysates were incubated with 0.1% Benzonase (Sigma-Aldrich) for 30 min at 4 °C and rotating at 20 rpm, centrifuged at 18,400*g* for 15 min, and the supernatants were collected. Protein concentrations were analysed by Bradford Protein Assay (Bio-Rad). Proteins were boiled in 1x NuPAGE LDS sample buffer (Invitrogen) with TCEP (Sigma-Aldrich), resolved on NuPAGE Novex 4–12% Bis-Tris gels (Invitrogen), and transferred onto nitrocellulose membranes (Bio-Rad) using Trans-Blot Turbo Transfer System (Bio-Rad). The membranes were blocked in PBS buffer with 0.1% Tween 20 and 5% non-fat dried milk for 1 h at room temperature and incubated overnight with primary antibodies at 4 °C, followed by 1 h incubation with peroxidase-conjugated secondary anti-mouse (Agilent, P0447, 1:2000) or anti-rabbit (Agilent, P0399, 1:2000) antibodies at room temperature. Blots were developed using ECL (Invitrogen) and analysed by exposing to films.

The following primary antibodies were used: mouse anti-BRCA1 (EMD Millipore, OP92, 1:1000), mouse anti-BRCA2 (EMD Millipore, OP95, 1:500), rabbit anti-tubulin (Abcam, ab6046, 1:10,000), rabbit anti-Pan ADPr binding reagent (EMD Millipore, MABE1016, 1:1500), rabbit anti-ARH3 (Atlas Antibodies, HPA027104, 1:1000), rabbit anti-γH2AX (Abcam, ab2893, 1:2000), rabbit anti-H2AX (Cell Signaling, 7631S, 1:1000), rabbit anti-H3 (EMD Millipore, 07-690, 1:50,000), rabbit anti-PARP1 (Abcam, ab32138, 1:5000), rabbit anti-H3S10P (Abcam, ab5176, 1:2000), rabbit anti-Poly/mono ADPr (Cell Signaling, 83732S, 1:1000), rabbit anti-HPF1 (DC Biosciences, custom-made against peptide RELPETDADLKRIC, 1:250 or custom-made published previously^27^, 1:1000), rabbit anti-GFP (Abcam, ab290, 1:5000) and mouse anti-FLAG (Sigma-Aldrich, A8592-1MG, 1:50,000).

### In vivo ADP-ribosylation assay

The 293T cells were transiently transfected with the indicated constructs using Polyfect Transfection Reagent (Qiagen) according to the Qiagen Quick-Start protocol or were left untransfected. For PARP1/PARP2 inhibitor titration experiments, 24 h after transfection, cells were treated with an inhibitor (Olaparib or Talazoparib) or equivalent amount of DMSO for 12 h. To induce DNA damage, the cells were washed once with DPBS and treated with 2 mM H_2_O_2_ in DPBS for 10 min, or treated with 0.1% MMS in complete DMEM for 30 min.

### In vitro ADP-ribosylation assay

Recombinant human HPF1 was expressed and purified as described previously and full-length PARP1 and PARP1 ΔHD as described for full-length PARP1^29^. Briefly, these proteins were expressed in Rosetta *Escherichia coli* cells with an N-terminal His_6_ tag and purified by Ni^2+^ affinity (both HPF1 and PARP1), followed by anion-exchange (HPF1) or heparin (PARP1) chromatography, and finally by size-exclusion chromatography using a Superdex 200 column (both HPF1 and PARP1). The last step was performed in the buffer 25 mM HEPES, pH 8, 200 mM NaCl, 1 mM EDTA and 0.1 mM TCEP. In the assay shown in Fig. 2d, 1 nM PARP1 ΔHD was incubated with 250 nM HPF1 (if indicated), 250 nM activating DNA duplex (5′-ATCAGATAGCATCTGTGCGGCCGCTTAGGG-3′ and 5′-CCCTAAGCGGCCGCACAGATGCTATCTGAT-3′), and indicated molarities of Olaparib in 50 mM Tris, pH 7.5, 50 mM NaCl. The reaction was started by adding ^32^P NAD^+^ from Perkin Elmer (50% cold at the time of use) to the final concentration of 62.5 nM, conducted for 3 min at room temperature, and quenched with SDS-containing gel-loading dye. The samples were analysed by SDS-PAGE and autoradiography. In the assay shown in Fig. 2e, 5 nM PARP1 was incubated with 2 µM HPF1 (if indicated), 2 µM recombinant human histone H1^0^ (New England BioLabs), and shown molarities of Olaparib in 50 mM Tris, pH 7.5, 50 mM NaCl. The reactions were started by adding 1 µM NAD^+^ and allowed to proceed for 3 min at room temperature before terminating with SDS-containing gel-loading dye. The results were analysed by SDS-PAGE and immunoblotting with the Pan ADPr reagent. Some non-specific recognition of unmodified H1 was observed.

### Binding analysis with fluorescence polarisation/anisotropy

Fluorescence polarisation/anisotropy experiments were performed in 25 mM HEPES, pH 7.2, 200 mM NaCl and 0.1 mM TCEP in black OptiPlate F 96-well plates (Perkin Elmer) at room temperature. The total sample volume was 100, 180 or 400 µl for *k*_on_ and *k*_off_ measurements and competition experiments, respectively. We used two different plate readers, SpectraMax M5 from Molecular Devices (settings: excitation/emission wavelengths of 485/530 nm; 20 readings per data point; and high PMT sensitivity) for *k*_off_ measurements and PHERAstar FS from BMG LabTech (settings: excitation/emission A/emission B wavelengths of 485/520/520 nm; 200 flashes/well; 0.5 s settling time; gain for A/B of 1571/1764; focal height adjusted) for *k*_on_ measurements and competition experiments. Data collection was performed using SoftMax Pro version 5.01 and PHERAstar software version 4.00 R3, respectively. For *k*_on_ measurements, samples containing 1 nM PARPi-FL (Tocris Bioscience), 3 µM HPF1 (if indicated) and 3 µM DNA duplex (the same as in the ADP-ribosylation assay) were mixed and their fluorescence polarisation measured. We began measuring time from this moment. Then, 1 µl of appropriate PARP1 dilution was rapidly mixed in and a time-course of 600 s with measurements every 10 s was started. In this set-up, HPF1 was not pre-incubated with PARP1, but we found that including HPF1, which, due to its high concentration, affects polarisation, in the sample prior to adding PARP1 was necessary for obtaining an accurate starting polarisation value. Since HPF1 is 3000x more concentrated than PARPi-FL, PARP1 is very likely saturated much faster with HPF1 than with the inhibitor. For *k*_off_ measurements, we pre-incubated 5 nM PARPi-FL with 25 nM PARP1, 3 µM HPF1 (if indicated), and 3 µM DNA duplex for 10 min prior to adding 164 µM non-fluorescent Olaparib. Polarisation was then monitored every 4 min over 19 h. For equilibrium competition experiments, we incubated 1 nM PARPi-FL with 6 nM PARP1, 3 µM HPF1 (if indicated), 0.5 µM duplex DNA, and a range of molarities of the indicated inhibitors for 20 h prior to measuring polarisation. For all experiments, the measured values were converted to anisotropy. Data from individual experiments were fitted to Eqs. (1), (2) or (3) (as appropriate) by minimising squared errors between actual and predicted values using Excel Solver:

1 $$-{\rm{association\kern-0.2pc:}}\,A={A}_{{\min }}+\left({A}_{{\max }}-{A}_{{\min }}\right)\times (1-{{\rm{e}}}^{-{k}_{{{\rm{on}}}}\times [{\rm{PARP}}1]\times t})$$

2 $$-{\rm{dissociation\kern-0.2pc:}}\,A={A}_{{\min }}+\left({A}_{{\max }}-{A}_{{\min }}\right)\times {{\rm{e}}}^{-{k}_{{{\rm{off}}}}\times t}$$

3 $$-{\rm{competition\kern-0.2pc:}}\,A={A}_{{\min }}+\frac{{A}_{{\max }}-{A}_{{\min }}}{1+{10}^{{{\log }}\left(\left[{\rm{Inhibitor}}\right]\right)-{{\log }}\left({{{\rm{IC}}}}_{50}\right)}},$$

where *A* is the measured anisotropy, *A*_min_ and *A*_max_ are the minimal and maximal anisotropy values (set to an empirical value or fitted as judged appropriate), [PARP1] is PARP1 concentration, *t* is time, *k*_on_ and *k*_off_ are association and dissociation rate constants, [Inhibitor] is inhibitor (Olaparib or Talazoparib) concentration, and IC_50_ is half-maximal inhibitory concentration. Each experimental repeat was fitted individually and then the mean and SD values calculated. *K*_D_ for the interaction between PARPi-FL and PARP1 was obtained using the formula: ${K}_{{\rm{D}}}=\frac{\bar{{k}_{{{\rm{on}}}}}}{\bar{{k}_{{{\rm{off}}}}}}$, and its SD with the formula (4):

4 $${\rm{SD}}=\frac{1}{\sqrt{N}}\frac{\bar{{k}_{{{\rm{on}}}}}}{\bar{{k}_{{{\rm{off}}}}}}\sqrt{\frac{{{{\rm{SD}}}_{{k}_{{{\rm{on}}}}}}^{2}}{{\bar{{k}_{{{\rm{on}}}}}}^{2}}+\frac{{{{\rm{SD}}}_{{k}_{{{\rm{off}}}}}}^{2}}{{\bar{{k}_{{{\rm{off}}}}}}^{2}}},$$

where $\bar{{k}_{{{\rm{on}}}}}$ and $\bar{{k}_{{{\rm{off}}}}}$ indicate the mean values of these rate constants and ${{\rm{SD}}}_{{k}_{{{\rm{on}}}}}$ and ${{\rm{SD}}}_{{k}_{{{\rm{off}}}}}$ represent the SD values of *k*_on_ and *k*_off_, respectively, while the sample size *N* = 3. The inhibitory constant *K*_i_ for Olaparib was estimated from IC_50_ as previously described^64^, using a Microsoft Excel file by Dr. Chao-Yie Yang available at http://www.umich.edu/~shaomengwanglab/software/calc_ki/index.html. This approach is unreliable for IC_50_ values close to, equal, or lower than the protein concentration used, precluding the estimation of *K*_i_ for Talazoparib.

### Live-cell imaging

Cells were seeded into an 8-well imaging chamber (Zell-Kontakt) and transfected with TagGFP2-PARP1 chromobody (Chromotek) and PATagRFP-H2B^65^ or GFP-PARP1 WT or point mutants and PATagRFP-H2B with X-tremeGene HP (Roche) according to the manufactures instructions 48 h prior to imaging. Cells were sensitised with fresh media containing 0.15 μg/mL Hoechst 33342 for 1 h at 37 °C. Immediately prior to imaging, the medium was replaced with CO_2_-independent phenol red-free Leibovitz’s L15 medium (Life Technologies) supplemented with 20% FBS, either with or without PARP1/PARP2 inhibitors. Cells were incubated with PARP inhibitors for a minimum of 30 min prior to imaging. Live-cell imaging was performed on a Nikon Ti-E inverted microscope equipped with a spinning-disk scan head CSU-X1 from Yokogawa at a rotation speed of 5000 rpm, a Plan APO 60x/1.4N.A. oil-immersion objective lens and a sCMOS ORCA Flash 4.0 camera. Laser microirradiation and local photoactivation at 405 nm was performed along a 16 µm line through the nucleus using a single-point scanning head (iLas2 from Roper Scientific) coupled to the epifluorescence backboard of the microscope. To ensure reproducibility, laser power at 405 nm was measured at the beginning of each experiment and set to 125 µW at the sample level. The fluorescence of GFP and PATagRFP were excited with lasers at 490 and 561 nm, respectively. For fluorescence detection, we used bandpass filters adapted to the fluorophore emission spectra. Cells were maintained at 37 °C with a heating chamber. Protein recruitment was quantified using custom-made MATLAB (MathWorks) routines available in a GitHub repository at https://github.com/sehuet/Recruitment-break. The characteristic dissociation time corresponds to the time required to dissipate 50% of the maximum PARP1 signal at the DNA lesion.

### Figure preparation

Graphs were prepared in Microsoft Excel 2016 or GraphPad PRISM 7 and further edited in Adobe Illustrator 25.1, which was also used to assemble all figures.

### Reporting summary

Further information on research design is available in the Nature Research Reporting Summary linked to this article.

## Supplementary information

Supplementary information.

Reporting summary.

## Data Availability

MATLAB routines used to quantify PARP1 recruitment in live-cell imaging experiments are deposited in a GitHub repository at https://github.com/sehuet/Recruitment-break. Any other relevant data are available from the authors. Source data are provided with this paper.

## Source data

Source Data
