# Supplementary material for: Serine-linked PARP1 auto-modification controls PARP inhibitor response
Source: Nat Commun. 2021 Jul 1;12:4055. doi: 10.1038/s41467-021-24361-9 (PMC8249464; doi:10.1038/s41467-021-24361-9)
Supplement: Supplementary file 1 — Supplementary information. [file 41467_2021_24361_MOESM1_ESM.pdf]

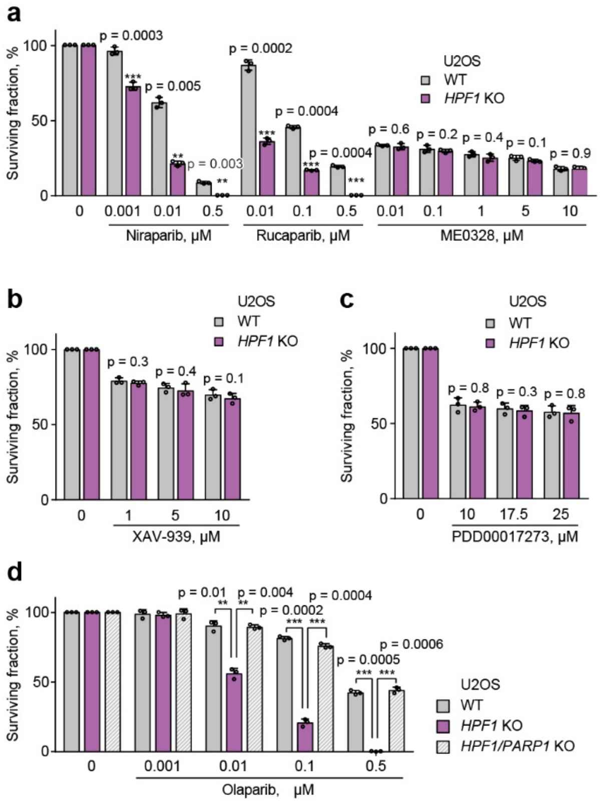

Supplementary Figure 1: Loss of HPF1 specifically sensitises cells to PARP1 or PARP2 inhibitors but not to inhibitors of other factors involved in the regulation of cellular ADPr levels (related to Fig. 1a).

a, Increased sensitivity of HPF1 KO cells to Olaparib treatment is abolished upon concurrent PARP1 KO. Significance of the difference between HPF1 KO and WT and HPF1/PARP1 KO and HPF1 KO is indicated. **b-d**, Reduced survival of HPF1 KO cells after treatment with PARP1/2 inhibitors Niraparib and Rucaparib but not with PARP3 inhibitor ME0328 (**b**), PARP5a/b inhibitor XAV-939 (**c**), or PARG inhibitor PDD00017273 (**d**). **a-d**, All data are shown as mean ± SD of three independent experiments. *, $p < 0.05$; **, $p < 0.01$; ***, $p < 0.001$ (two-tailed Student's t-test).

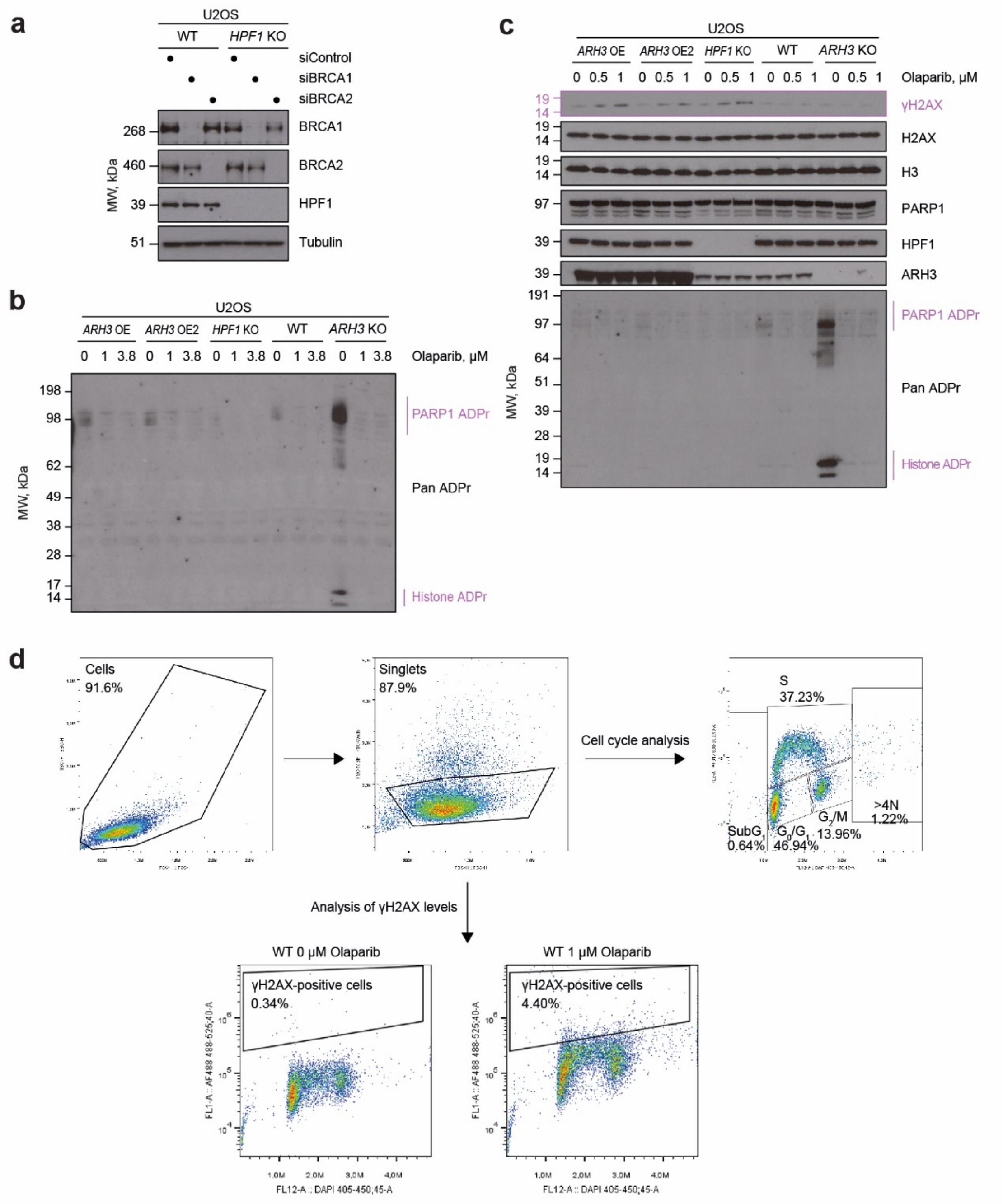

Supplementary Figure 2: HPF1 and ARH3 status affects cell ADPr levels, Olaparib-induced γH2AX formation and changes in cell cycle distribution (related to Fig. 1b-e).

a, siRNAs against *BRCA1* and *BRCA2* specifically downregulate these genes in U2OS WT and HPF1 KO cells. These are control immunoblots to accompany **Fig. 1b**. **b**, Pan ADPr immunoblot for the experiment in **Fig. 1c**. **c**, A repeat of the experiment in **Fig. 1c** performed with lower Olaparib concentration used for a 6-day treatment. Olaparib-induced *γ*H2AX formation depends on cellular HPF1 and ARH3 protein levels. **d**, Flow cytometry gating strategy for analysis of cell cycle distribution in **Fig. 1d** and **e** and γH2AX levels in **Fig. 1e**. The experiments in **a-c** were repeated independently at least two times with similar results.

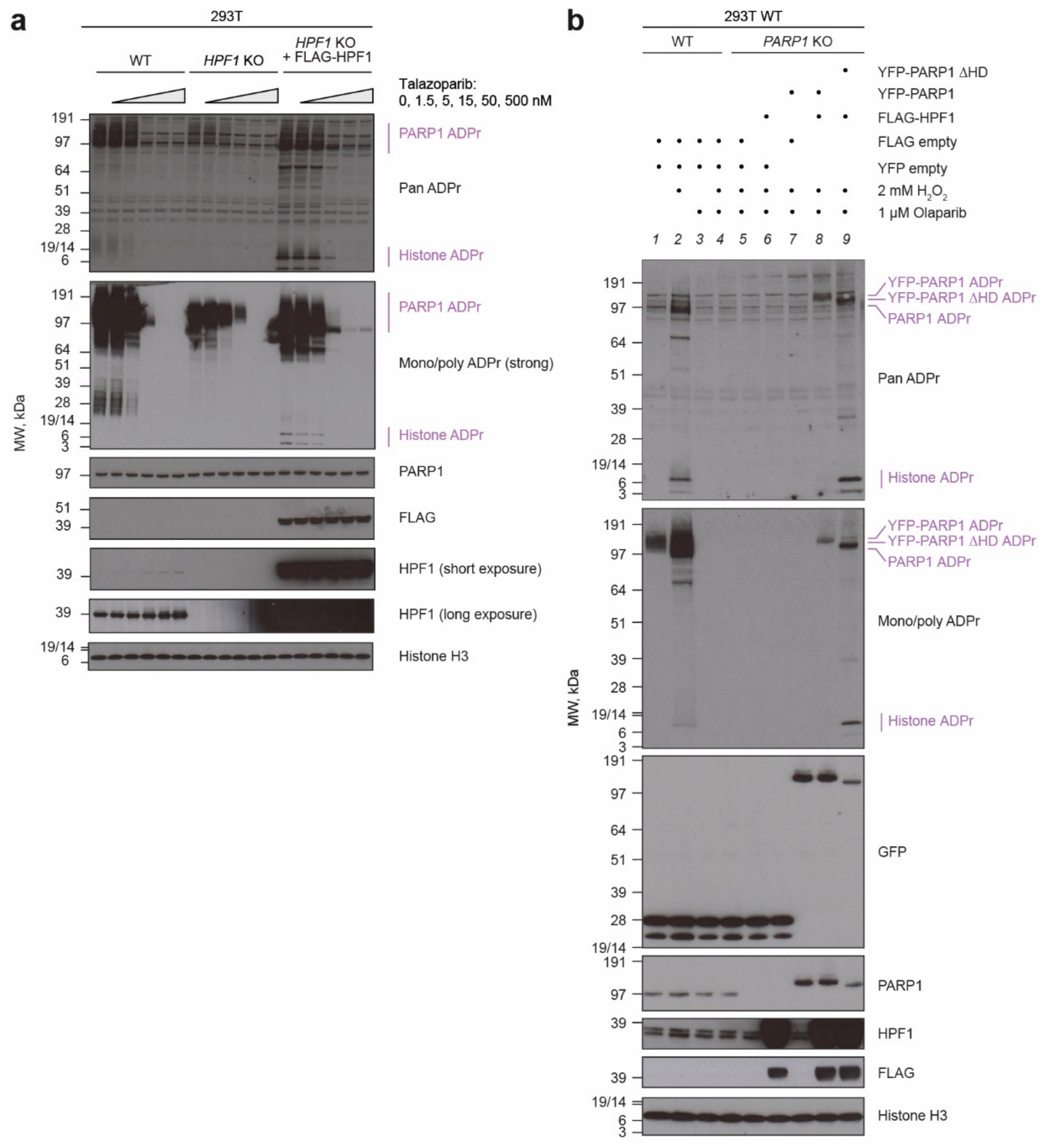

Supplementary Figure 3: Persistence of HPF1-dependent ADP-ribosylation signal at elevated PARP1/PARP2 inhibitor concentrations (related to Fig. 2a, c).

a, Cellular ADPr levels detected throughout increasing molarities of Talazoparib depend on HPF1 status. The effects of 12-h Talazoparib treatment on cells with endogenous HPF1 levels, loss or overexpression of HPF1 are compared. **b**, Simultaneous DNA damage (H_2O_2 treatment) and HPF1 overexpression stimulate PARP1 WT and ΔHD to produce ADPr that is detectable at a high Olaparib concentration. The experiments in **a** and **b** were repeated at least two times with similar results.

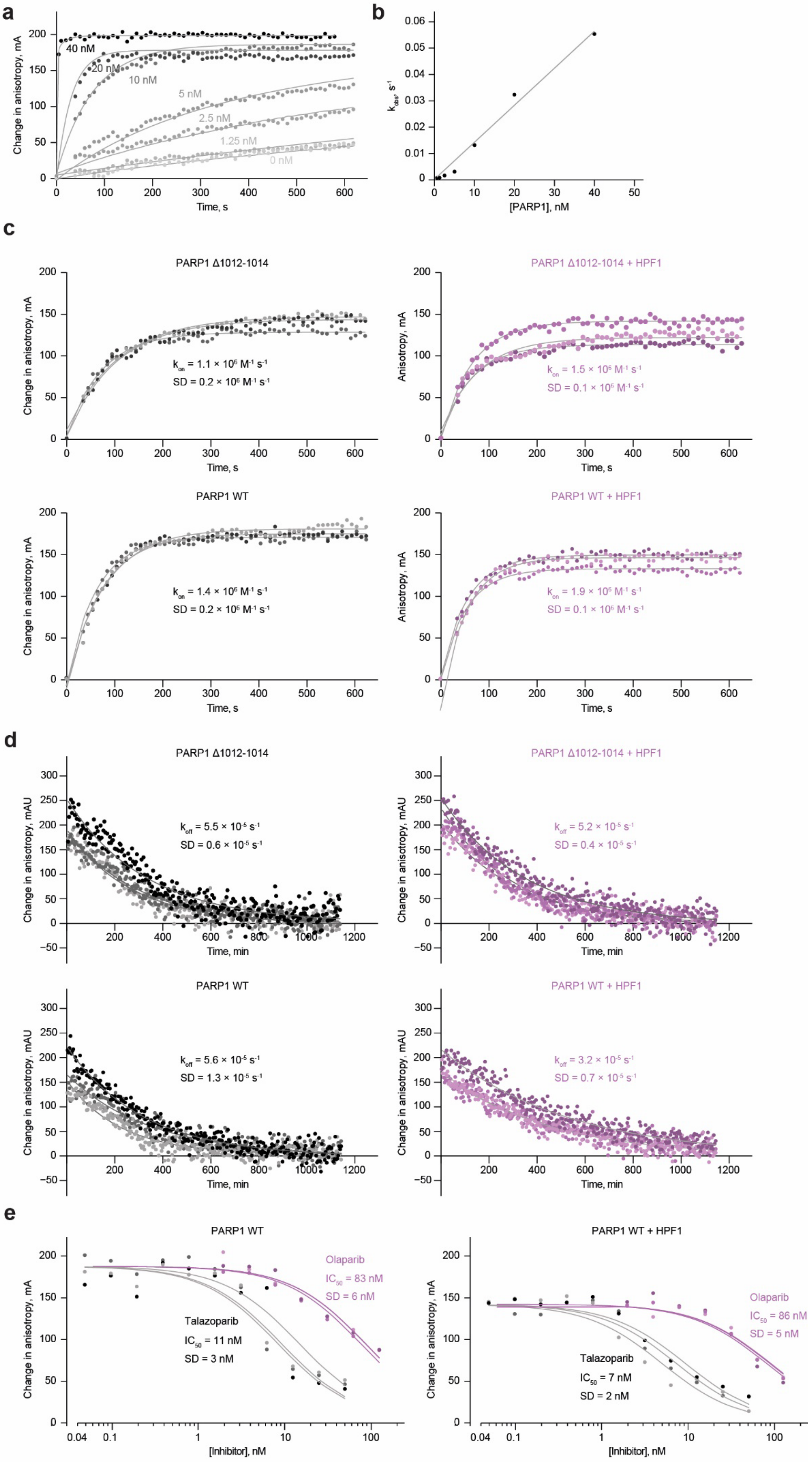

Supplementary Figure 4: Binding of PARP inhibitors to PARP1 in the presence or absence of HPF1.

a-c, Measuring association between the fluorescent Olaparib-derivative PARPi-FL and PARP1 using fluorescence anisotropy. Association of PARPi-FL with increasing molarities of PARP1 (**a**) was analysed, showing linear dependence of the apparent association rate constant k_{obs} on PARP1 concentration (**b**). PARP1 concentration of 10 nM was chosen for subsequent comparison of PARPi-FL binding to WT or HPF1 binding-deficient (Δ1012-1014) PARP1 in the presence or absence of HPF1 (**c**). **d**, Measuring dissociation of PARPi-FL from PARP1 (WT or Δ1012-1014) in the presence or absence of HPF1 after addition of a large excess of unlabelled Olaparib. **e**, Equilibrium competition assay in which PARPi-FL was outcompeted for PARP1 binding with increasing molarities of Talazoparib or Olaparib. The scale on the horizontal axis is logarithmic. For each of the experiments **c-e**, three repeats (different samples) were performed and fitted individually to estimate the association and dissociation rate constants (k_{on} and k_{off}) or the half-maximal inhibitory concentration (IC_{50}) as described in Methods. The mean and SD of these estimates are provided. More extensive data quantification and processing are shown in **Supplementary Tables 1 and 2**.

Supplementary Table 1: PARPi-FL binding to WT or HPF1 binding-deficient (Δ1012-1014) PARP1 in the presence or absence of HPF1

	k_{on} (10^6 M^{-1} s^{-1})*	k_{off} (10^{-5} s^{-1})*	K_D (pM)**
PARP1 WT	1.4 ± 0.2	5.6 ± 1.3	39 ± 10
PARP1 WT + HPF1	1.9 ± 0.1	3.2 ± 0.7	17 ± 3
PARP1 Δ1012-1014	1.1 ± 0.2	5.5 ± 0.6	51 ± 14
PARP1 Δ1012-1014 + HPF1	1.5 ± 0.1	5.2 ± 0.4	36 ± 6

* Data from each of three technical repeats (**Supplementary Fig. 4c** and **d**) were fitted as described in Methods. Mean ± SD of the values obtained from the fitting are presented.

** Calculated from k_{on} and k_{off} as described in Materials and methods.

Supplementary Table 2: Olaparib and Talazoparib binding to PARP1 in the presence and absence of HPF1 estimated by outcompeting PARPi-FL

	IC_{50} (nM)*	K_i estimate (nM)**
Talazoparib	11 ± 3	Not reliable
Talazoparib (+ HPF1)	7 ± 2	Not reliable
Olaparib	83 ± 6	0.54
Olaparib (+ HPF1)	86 ± 5	0.25

* Refers to the amount of inhibitor needed to outcompete 1 nM PARPi-FL for binding to 6 nM PARP1. Data from each of three technical repeats (**Supplementary Fig. 4e**) were fitted as described in Methods. Mean ± SD of the values obtained from the fitting are presented.

** Calculated as described in Methods from the mean IC_{50} values and the K_D values for PARPi-FL from **Supplementary Table 1**.

Supplementary Table 3: Primer sequences

Primers used for site-directed mutagenesis of PARP1 constructs. To achieve mutations of multiple sites, the appropriate mutations were introduced sequentially.

Mutation	Forward primer	Reverse primer
S499A	PARP1_T1495G_SENSE: CCAAGAGGGAAGGCAGGGGCTGCGC	PARP1_T1495G_ANTISENSE: GCGCAGCCCCTGCCTTCCCTCTTGG
S507A	PARP1_A1519G_G1520C_SENSE: GGCTGCGCTCTCCAAAAAAGCCAAGGGCCAGG	PARP1_A1519G_G1520C_ANTISENSE: CCTGGCCCTTGGCTTTTTTGGAGAGCGCAGCC
S519A	PARP1_T1555G_SENSE: GTTAATTTCATTCTCTTTTCAGCTTTGTTGATACCTTCCTCCTTGA	PARP1_T1555G_ANTISENSE: TCAAGGAGGAAGGTATCAACAAAGCTGAAAAGAGAATGAAATTAAC
Y634A	357_PARP1_Y634A_FOR: AGGGGGTAGAACTTTTTGGGAGCCTTCGTGAAATTTTTGGAGTG	358_PARP1_Y634A_REV: CACTCCAAAAATTTCACGAAGGCTCCCAAAAAGTTCTACCCCCT
E471A	PARP1_ A1412C_SENSE: GTGCGCTAAGAACAACGCCTGAAGGCTCTTGGT	PARP1_ A1412C_ANTISENSE: ACCAAGAGCCTTCAGGCGTTGTTCTTAGCGCAC
E484A	PARP1_A1451C_SENSE: CTCTGCCTTCACCGCTGCCCCCCAAGG	PARP1_A1451C_ANTISENSE: CCTTGGGGGGCAGCGGTGAAGGCAGAG
A488/491A	PARP1_A1463C_A1472C_SENSE: GGGGCCACAACTGCAACAGGCGCTGCCTTCACCT	PARP1_A1463C_A1472C_ANTISENSE: AGGTGAAGGCAGCGCCTGTTGCAGTTGTGGCCCC
E513/514A	PARP1_A1538C_A1541C_SENSE: CAGATTTGTTGATACCTGCCGCCTTGACCTGGCCCTTG	PARP1_A1538C_A1541C_ANTISENSE: CAAGGGCCAGGTCAAGGCGGCAGGTATCAACAAATCTG

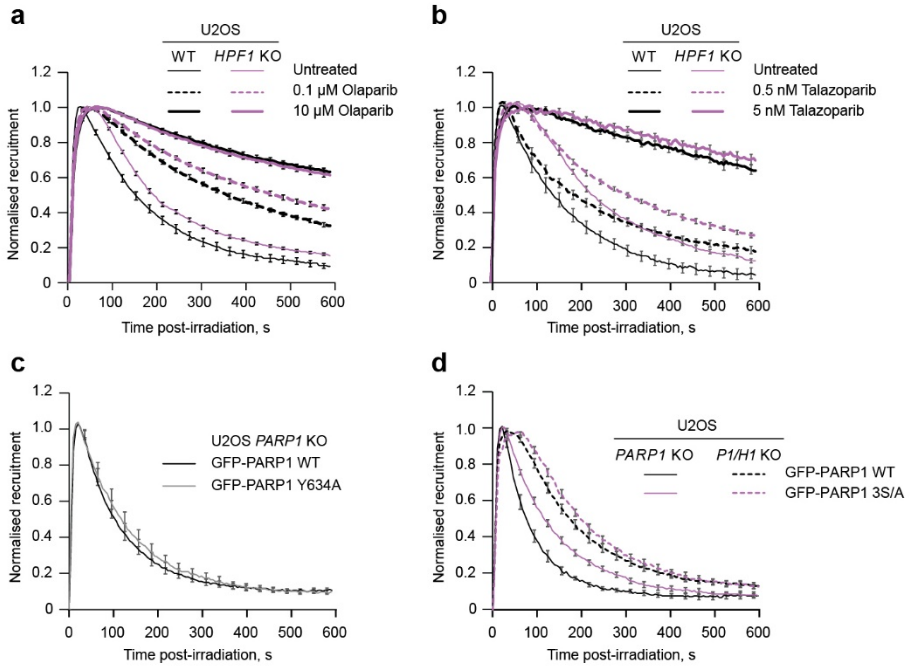

Supplementary Figure 5: PARP1 recruitment to sites of laser microirradiation depends on HPF1, PARP1 activity and PARP1 auto-modification on serine but not tyrosine sites (related to Fig. 3).

a-b, Quantification of GFP-PARP1 chromobody at sites of laser microirradiation in WT and HPF1 KO cells in the presence of indicated molarities of Olaparib (**a**) or Talazoparib (**b**). **c**, Quantification of GFP-PARP1 WT or PARP1 Y634A at damaged sites in PARP1 KO cells. **d**, Quantification of GFP-PARP1 WT or 3S/A at damaged sites in PARP1 KO or PARP1/HPF1 KO (P1/H1 KO) cells. For **a-d**, individual curves representing PARP1 recruitment kinetics at damage sites were normalized to maximum recruitment. All data are shown as mean ± SEM from the analysis of at least 10 nuclei.

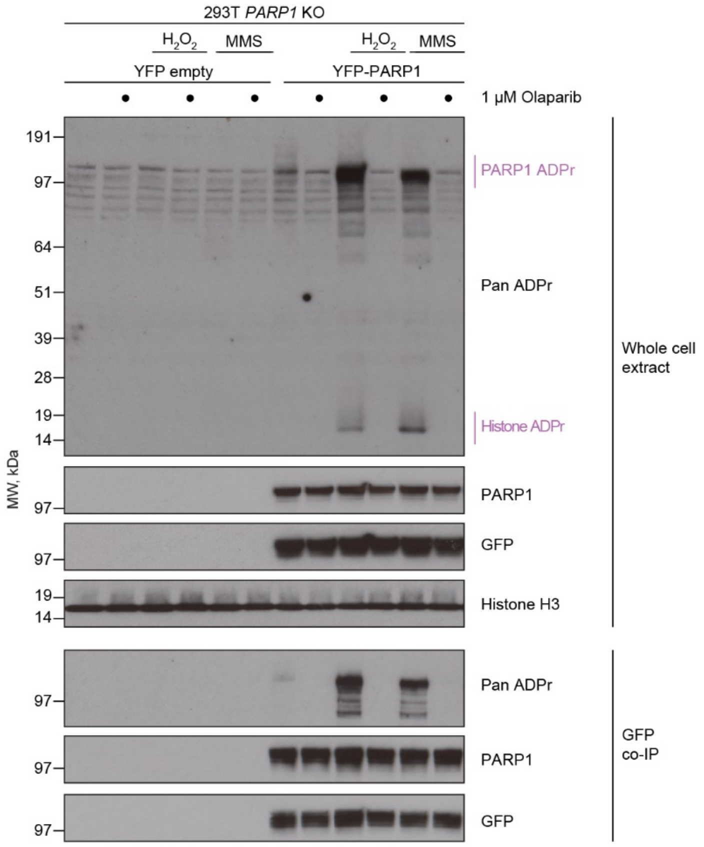

Supplementary Figure 6: DNA damaging agents H_2O_2 and MMS trigger similar patterns of cellular ADPr (related to Fig. 3).

Immunoblots of whole cell extract and GFP coimmunoprecipitation (GFP co-IP) samples are shown. ADPr is localised primarily to PARP1 and histones. The experiment was repeated independently three times with similar results.

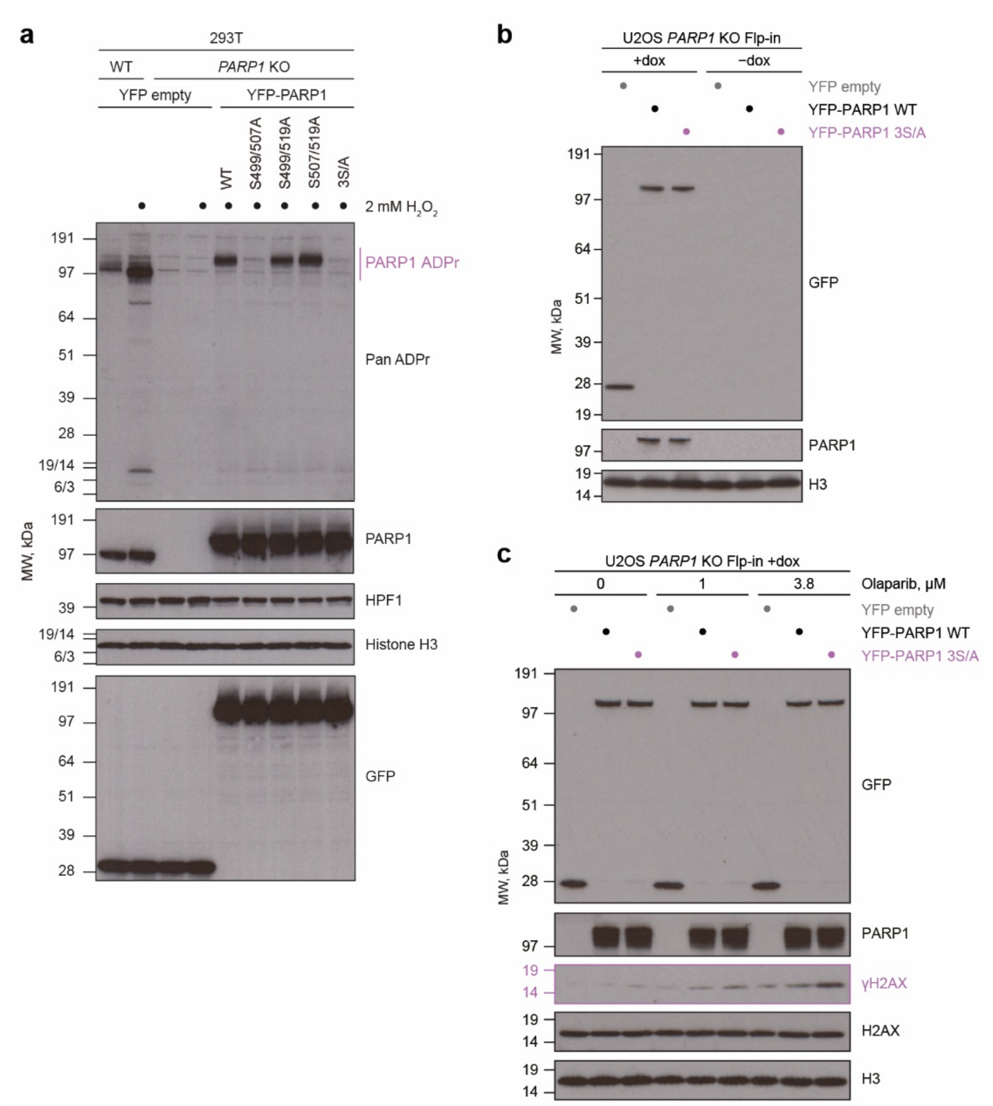

Supplementary Figure 7: S499, S507 and, to a lesser extent, S519 are the major sites of PARP1 auto-modification, which loss leads to increased γH2AX formation upon Olaparib treatment (related to Fig. 3).

a, Comparison of the effect of PARP1 S499/507/519A (3S/A), S499/507A, S499/519A and S507/519A mutations on DNA damage (H_2O_2)-induced ADPr levels. **b**, Control immunoblots comparing expression of GFP-PARP1 WT and 3S/A to accompany **Fig. 3i**. **c**, Loss of key serine auto-modification sites in PARP1 3S/A mutant leads to increased γH2AX formation following 6-day Olaparib treatment. The experiments in **a**-**c** were repeated independently two times with similar results.
