# Supplementary material for: Serine-linked PARP1 auto-modification controls PARP inhibitor response
Source: Nat Commun. 2021 Jul 1;12:4055. doi: 10.1038/s41467-021-24361-9 (PMC8249464; doi:10.1038/s41467-021-24361-9)
Supplement: Supplementary file 2 — Reporting summary. [file 41467_2021_24361_MOESM2_ESM.pdf]

nature research

Corresponding author(s): Ivan Ahel and Marcin J. Suskiewicz

Last updated by author(s): Jun 7, 2021

Reporting Summary

Nature Research wishes to improve the reproducibility of the work that we publish. This form provides structure for consistency and transparency in reporting. For further information on Nature Research policies, see our Editorial Policies and the Editorial Policy Checklist.

Statistics

For all statistical analyses, confirm that the following items are present in the figure legend, table legend, main text, or Methods section.

n/a	Confirmed	
☐	☒	The exact sample size (*n*) for each experimental group/condition, given as a discrete number and unit of measurement
☐	☒	A statement on whether measurements were taken from distinct samples or whether the same sample was measured repeatedly
☐	☒	The statistical test(s) used AND whether they are one- or two-sided<br>*Only common tests should be described solely by name; describe more complex techniques in the Methods section.*
☒	☐	A description of all covariates tested
☒	☐	A description of any assumptions or corrections, such as tests of normality and adjustment for multiple comparisons
☐	☒	A full description of the statistical parameters including central tendency (e.g. means) or other basic estimates (e.g. regression coefficient) AND variation (e.g. standard deviation) or associated estimates of uncertainty (e.g. confidence intervals)
☐	☒	For null hypothesis testing, the test statistic (e.g. *F*, *t*, *r*) with confidence intervals, effect sizes, degrees of freedom and *P* value noted<br>*Give P values as exact values whenever suitable.*
☒	☐	For Bayesian analysis, information on the choice of priors and Markov chain Monte Carlo settings
☒	☐	For hierarchical and complex designs, identification of the appropriate level for tests and full reporting of outcomes
☒	☐	Estimates of effect sizes (e.g. Cohen's *d*, Pearson's *r*), indicating how they were calculated

Our web collection on statistics for biologists contains articles on many of the points above.

Software and code

Policy information about availability of computer code

Data collection	Flow cytometry data were collected using CytExpert Acquisition and Analysis Software Version 2.3 (Beckman Coulter). Data collection for fluorescence polarisation/anisotropy assays was performed using SoftMax Pro version 5.01 and PHERAstar software version 4.00 R3.
Data analysis	Flow Cytometry data were analysed using FlowJo v10 (BD Biosciences). Live-cell imaging data were analysed using Matlab (MathWorks). Image analysis for the colony formation assay was done using ImageJ.<br>Data visualization and statistical analysis was done using GraphPad PRISM 7, Microsoft Excel 2016 (including Solver). Images were assembled in Adobe Illustrator 25.1.

For manuscripts utilizing custom algorithms or software that are central to the research but not yet described in published literature, software must be made available to editors and reviewers. We strongly encourage code deposition in a community repository (e.g. GitHub). See the Nature Research guidelines for submitting code & software for further information.

Data

Policy information about availability of data

All manuscripts must include a data availability statement. This statement should provide the following information, where applicable:

- Accession codes, unique identifiers, or web links for publicly available datasets
- A list of figures that have associated raw data
- A description of any restrictions on data availability

All raw data are included in the manuscript or can be provided upon request.

April 2020

Field-specific reporting

Please select the one below that is the best fit for your research. If you are not sure, read the appropriate sections before making your selection.

☒ Life sciences ☐ Behavioural & social sciences ☐ Ecological, evolutionary & environmental sciences

For a reference copy of the document with all sections, see nature.com/documents/nr-reporting-summary-flat.pdf

Life sciences study design

All studies must disclose on these points even when the disclosure is negative.

Sample size	No statistical method was used to determine sample size as no inference from small sample to larger populations was done. Two or three independent repeats were performed for each experiment to confirm reproducibility according to the best practice in the field.
Data exclusions	No data were excluded.
Replication	All experiments were repeated at least two to three times independently with similar results. All observations were found to be reproducible.
Randomization	Randomisation was not required due to the mechanistic character of the study with objective readouts and lack of animal-based experiments.
Blinding	No blinding was performed as the bias from prior knowledge of samples in case of biochemical, blotting, survival, and imagining experiments described in the study is considered limited. All samples including controls were analysed in exactly the same manner.

Reporting for specific materials, systems and methods

We require information from authors about some types of materials, experimental systems and methods used in many studies. Here, indicate whether each material, system or method listed is relevant to your study. If you are not sure if a list item applies to your research, read the appropriate section before selecting a response.

Materials & experimental systems

n/a	Involved in the study	
☐	☒	Antibodies
☐	☒	Eukaryotic cell lines
☒	☐	Palaeontology and archaeology
☒	☐	Animals and other organisms
☒	☐	Human research participants
☒	☐	Clinical data
☒	☐	Dual use research of concern

Methods

n/a	Involved in the study	
☒	☐	ChIP-seq
☐	☒	Flow cytometry
☒	☐	MRI-based neuroimaging

Antibodies

Antibodies used	The following primary antibodies were used for immunoblotting:<br>commercial mouse anti-BRCA1 (EMD Millipore, OP92, 1:1,000), mouse anti-BRCA2 (EMD Millipore, OP95, 1:500), rabbit anti-tubulin (Abcam, ab6046, 1:10,000), rabbit pan-ADPr binding reagent (EMD Millipore, MABE1016, 1:1,500), rabbit anti-ARH3 (Atlas Antibodies, HPA027104, 1:1,000), rabbit anti-γH2AX (Abcam, ab2893, 1:2,000), rabbit anti-H2AX (Cell Signaling, 7631S, 1:1,000), rabbit anti-H3 (EMD Millipore, 07-690, 1:50,000), rabbit anti-PARP1 (Abcam, ab32138, 1:5,000), rabbit anti-H3S10P (Abcam, ab5176, 1:2,000), rabbit anti-mono/poly ADPr (Cell Signaling, 83732, 1:1,000), rabbit anti-GFP (Abcam, ab290, 1:5,000), mouse anti-Flag (Sigma Aldrich, A8592-1MG, 1:50,000); custom-made rabbit anti-HPF1 (Gibbs-Seymour et al., 2016, 1:1,000), rabbit anti-HPF1 (DC Biosciences, against peptide RELPETDADLKRIC, 1:250).<br>The following secondary antibodies were used for immunoblotting:<br>goat anti-mouse (Agilent, P0447, 1:2,000), swine anti-rabbit (Agilent, P0399, 1:2,000).<br>Rabbit anti-γH2AX primary antibody (Cell Signaling, 9718S, 1:200) and Alexa Fluor 488-conjugated goat anti-rabbit secondary antibody (Molecular Probes/Thermo Fisher, A11034, 1:500) were used for flow cytometry.
Validation	All commercial antibodies were validated by manufacturers for the use at least in immunoblotting and immunoprecipitation. The commercial anti-HPF1 anti-body was validated by the Human Priotein Atlas project in multiple tissues and cell lines (data at https://www.proteinatlas.org/ENSG00000056050-HPF1) and additionally by us using immunoblotting with 293T WT and HPF1 KO cells (Suskiewicz et al., Nature, 2020). The anti-ARH3 antibody was validated by the manufacturer using both immunoblotting and immunocytochemistry (images at https://www.atlasantibodies.com/products/antibodies/primary-antibodies/triple-a-polyclonals/adprhl2-antibody-hpa027104/) and addtionally by us using immunoblotting in 293T and U2OS WT and ARH3 KO cell lines (see Palazzo et al., eLife, 2018). The custom-made anti-HPF1 antibody was validated by us with immunoblotting using purified human HPF1 and 293T and U2OS WT and HPF1 KO cells (Gibbs-Seymour et al., Mol Cell, 2016 and Palazzo et al., eLife, 2018), as well as testis, ovary, brain, heart, kidney and liver tissue from WT and HPF1 KO mice (unpublished). Anti γ-H2AX antibody was validated by the manufacturer for the use in immunocytochemistry and immunoblotting (see data at https://www.abcam.com/gamma-

h2axphospho-
s139-antibody-ab2893.html) and additionally by us for the use in flow cytometry by comparing untreated cells and cells treated with genotoxic drugs. Anti pan-ADPr binding reagent (MABE1016) was extensively validated with dot blot, immunoblotting, and immunofluorescence by the Krauss group as described in Gibson et al., Biochemistry, 2017.

Eukaryotic cell lines

Policy information about cell lines

Cell line source(s)	293T (CRL-3216) and U2OS (HTB-96) cells were obtained from ATCC. U2OS Flp-In T-Rex cells were a gift from Fumiko Esashi (University of Oxford), but they come from Daniel Durocher's lab who described their generation in Stewart et al., Cell, 2009.
Authentication	Cells were not authenticated in our lab. 293T and U2OS cell lines were authenticated with morphology, karyotyping, and PCR based approaches by ATCC.
Mycoplasma contamination	The cell lines were tested for mycoplasma contamination and confirmed as mycoplasma negative.
Commonly misidentified lines (See ICLAC register)	No commonly misidentified lines were used.

Flow Cytometry

Plots

Confirm that:

☒ The axis labels state the marker and fluorochrome used (e.g. CD4-FITC).

☒ The axis scales are clearly visible. Include numbers along axes only for bottom left plot of group (a 'group' is an analysis of identical markers).

☒ All plots are contour plots with outliers or pseudocolor plots.

☒ A numerical value for number of cells or percentage (with statistics) is provided.

Methodology

Sample preparation	For cell cycle analysis, human U2OS osteosarcoma (ATCC HTB-96) cells were seeded in 6-well plates, treated and incubated with 10 µM EdU for 1 hour at the end of treatment. Cells were harvested by trypsinization and labelled using the Click-iT Plus EdU Alexa Fluor 647 Flow Cytometry Assay Kit (Invitrogen) according to the manufacturer's instructions. For the analysis of DSB levels, cells were then stained protected from light with γH2AX primary antibody (Cell Signaling, 9718S, 1:200) in 1% BSA in PBS for 1 hour at room temperature, washed once and incubated for 30 minutes with Alexa Fluor 488-conjugated goat anti-rabbit secondary antibody (Molecular Probes/Thermo Fisher, A11034, 1:500) in 1% BSA in PBS. For DAPI staining, cell pellets were resuspended in 1 µg/mL DAPI solution in PBS and incubated for 10 minutes. Cells were washed in PBS and analysed immediately after staining.
Instrument	Cytoflex LX (Beckman Coulter).
Software	Data were collected using CytExpert Acquisition and Analysis Software Version 2.3 (Beckman Coulter) and analysed using FlowJo v10 (BD Biosciences).
Cell population abundance	Cells were not sorted in this study.
Gating strategy	Cells were first selected by excluding events with low forward (FSC) and side scatter (SSC) signals. Doublets or aggregates were excluded after plotting FSC or SSC Area versus Height or Width. Cell cycle analysis was performed on the single cell population after plotting EdU intensity (Alexa Fluor 647) against DAPI signal. γH2AX-positive and -negative single cells were discriminated based on the increase in γH2AX signal in Olaparib-treated samples as compared to untreated cells after plotting γH2AX intensity (Alexa Fluor 488) against DAPI.

☒ Tick this box to confirm that a figure exemplifying the gating strategy is provided in the Supplementary Information.
